# Poor nutritional quality of primary producers and zooplankton driven by eutrophication is mitigated at upper trophic levels

**DOI:** 10.1002/ece3.8687

**Published:** 2022-03-08

**Authors:** Sami Johan Taipale, Anne‐Mari Ventelä, Jaakko Litmanen, Lauri Anttila

**Affiliations:** ^1^ Department of Biological and Environmental Science University of Jyväskylä Jyväskylä Finland; ^2^ Pyhäjärvi Institute Ruukinpuisto Kauttua Finland

**Keywords:** benthic invertebrates, freshwater food web, ontogenetic diet shift, perch, phytoplankton, polyunsaturated fatty acids

## Abstract

Eutrophication and rising water temperature in freshwaters may increase the total production of a lake while simultaneously reducing the nutritional quality of food web components. We evaluated how cyanobacteria blooms, driven by agricultural eutrophication (in eutrophic Lake Köyliöjärvi) or global warming (in mesotrophic Lake Pyhäjärvi), influence the biomass and structure of phytoplankton, zooplankton, and fish communities. In terms of the nutritional value of food web components, we evaluated changes in the ω‐3 and ω‐6 polyunsaturated fatty acids (PUFA) of phytoplankton and consumers at different trophic levels. Meanwhile, the lakes did not differ in their biomasses of phytoplankton, zooplankton, and fish communities, lake trophic status greatly influenced the community structures. The eutrophic lake, with agricultural eutrophication, had cyanobacteria bloom throughout the summer months whereas cyanobacteria were abundant only occasionally in the mesotrophic lake, mainly in early summer. Phytoplankton community differences at genus level resulted in higher arachidonic acid, eicosapentaenoic acid (EPA), and docosahexaenoic acid (DHA) content of seston in the mesotrophic than in the eutrophic lake. This was also reflected in the EPA and DHA content of herbivorous zooplankton (*Daphnia* and *Bosmina*) despite more efficient trophic retention of these biomolecules in a eutrophic lake than in the mesotrophic lake zooplankton. Planktivorous juvenile fish (perch and roach) in a eutrophic lake overcame the lower availability of DHA in their prey by more efficient trophic retention and biosynthesis from the precursors. However, the most efficient trophic retention of DHA was found with benthivorous perch which prey contained only a low amount of DHA. Long‐term cyanobacterial blooming decreased the nutritional quality of piscivorous perch; however, the difference was much less than previously anticipated. Our result shows that long‐term cyanobacteria blooming impacts the structure of plankton and fish communities and lowers the nutritional quality of seston and zooplankton, which, however, is mitigated at upper trophic levels.

## Graphical abstract

We evaluated how cyanobacteria blooms driven by agricultural eutrophication and global warming influence on the biomass and structure of phytoplankton, zooplankton, and fish communities and nutritional value of different food web components. Our result showed that lower availability of physiologically important long‐chain PUFA by primary producers and at lower trophic levels is mitigated by efficient trophic retention and biosynthesis at upper trophic levels.

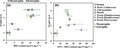

## 1 INTRODUCTION

Globally, freshwater ecosystems are challenged by land use and many factors connected to climate warming, such as changing precipitation, eutrophication (as an increase in total phosphorus) (Hasler, 1947), and water browning (an increase of DOC) (Karlsson et al., 2009; Leech et al., 2018; O'Reilly et al., 2003). In the boreal zone, lake water temperature and precipitation are increasing, which may increase nitrogen (N), phosphorus (P), and dissolved organic carbon (DOC) loading, especially from agricultural and peatland‐dominated catchments (Lathrop et al., 2019; Ruosteenoja et al., 2016). Changing environmental conditions affect ecosystem function and phytoplankton, zooplankton, and fish community structure (Havens, 2008; Jeppesen et al., 2010, 2012; Keva et al., 2021; Sukenik et al., 2015; Ventelä et al., 2016). At the same time, these conditions also impact the nutritional value of the phytoplankton and thus the production and the transfer of essential biomolecules through food webs (Lau et al., 2021; Müller‐Navarra et al., 2004; Taipale et al., 2016, 2019).

The ω‐3 and ω‐6 polyunsaturated fatty acids (PUFA) have been found to have many physiologically necessary functions in all animals including humans (Arts et al., 2009; Simopoulos, 2000). Because animals cannot synthesize ω‐3 and ω‐6 PUFA de novo, they need to obtain these molecules from their diet. Therefore, short‐chain ω‐3 and ω‐6 PUFA of α‐linolenic acid (ALA, 18:3ω3) and linoleic acid (LA, 18:2ω6) are usually considered essential fatty acids (EFA) or “essential nutrients” for animals (Parrish, 2009). However, eicosapentaenoic acid (EPA, 20:5ω3), docosahexaenoic acid (DHA, 22:6ω3), and arachidonic acid (ARA, 20:4ω6) are physiologically most important for consumers (Hulbert & Abbott, 2012; Parrish, 2009; Stanley‐Samuelson et al., 1988). Therefore, they may be called physiologically essential or semi‐essential PUFA (Taipale et al., 2019).

In marine and freshwater ecosystems, green algae and cyanobacteria are classified as non‐EPA and non‐DHA‐synthesizers, while golden algae, dinoflagellates, cryptophytes, diatoms, and raphidophytes are primary producers of EPA and DHA (Ahlgren et al., 1992; Jónasdóttir, 2019; Taipale et al., 2013; Taipale, Vuorio, et al., 2016). However, EPA‐ and DHA‐ synthesizing phytoplankton taxa can also be found abundantly in eutrophic lakes (Lepistö & Rosenström, 1998). A clear decline in the nutritional quality of seston can be seen in hyper‐eutrophic lakes (Müller‐Navarra et al., 2004; Taipale et al., 2019). Therefore, it is important to monitor the abundance of EPA‐ and DHA‐synthesizing phytoplankton taxa (cryptomonads, golden algae, diatoms, dinoflagellates, raphidophytes, euglenoids) throughout the summer to understand the nutritional quality of phytoplankton. Agricultural eutrophication has been the main reason for increased cyanobacteria blooms in boreal and temperate lakes (Jørgensen & Rast, 2001). However, the growing abundance of cyanobacteria blooms in the recent past is related to climate change and especially due to the increased temperature of lakes (Deng et al., 2016; Elliot, 2012; Paerl & Huisman, 2008; Pätynen et al., 2014; Rasconi et al., 2017). Previous studies have shown that a decrease in the nutritional quality of phytoplankton is mainly attributed to the changes by the phytoplankton community structure, but also because the nutritional value of phytoplankton cells decreases by eutrophication (Keva et al., 2021; Lau et al., 2021; Taipale et al., 2019).

Herbivorous zooplankton is a key link in connecting phytoplankton and planktivorous fish and thus the nutritional value of zooplankton is important for the growth of fish fry (Taipale et al., 2018). However, individual zooplankton taxa differ by their nutritional value (Kratina & Winder, 2015). This is because cladocerans usually accumulate EPA whereas copepods are rich in DHA (Brett et al., 2009; Hiltunen et al., 2016; Smyntek et al., 2008; Taipale et al., 2011). Moreover, zooplanktons are generally inefficient in their ability to biosynthesize ALA to EPA and DHA. Thus, they are strongly dependent on the fatty acid quality in their diet (Elert et al., 2003; Koussoroplis et al., 2014; Taipale et al., 2011). Herbivorous cladoceran (*Daphnia* and *Bosmina*) is a keystone species in most lake ecosystems (Bergquist et al., 1985; Kerfoot et al., 1988; Lynch & Shapiro, 1981). It can detect high nutritional quality patches and can selectively feed on high nutritional quality particles (Hartmann & Kunkel, 1991; Schatz & McCauley, 2007). Moreover, fatty acid‐based modeling has shown that seston microbial (including algae) composition does not necessarily match with assimilated diet (Taipale et al., 2019). This is because herbivorous zooplankton (*Daphnia* and *Bosmina*) favors high nutritional quality diet (Galloway et al., 2014; Taipale et al., 2019). Cyanobacteria blooms may lead to poorer energy flow in aquatic food webs because they poorly support zooplankton somatic growth and reproduction (Bednarska et al., 2014; Elert et al., 2003; Peltomaa et al., 2017; Porter & McDonough, 1984). They can also be linked to the upper trophic level only by certain zooplankton taxa (e.g., *Chydorus*; Tõnno et al., 2016). Environmental changes (e.g., eutrophication, browning, global warming) have been shown to have different impacts on the nutritional value of zooplankton (Keva et al., 2021; Lau et al., 2021; Senar et al., 2019). As a result, it appears that the lower nutritional value of phytoplankton does not always affect higher trophic levels. However, a recent study of productivity and temperature gradient in sub‐arctic lakes showed that the zooplankton community changed from the Calanoid (*Eudiaptomus graciloides*) dominated community towards herbivorous cladocerans (*Daphnia* and *Bosmina*), resulting in a decrease in the EPA and DHA content of zooplankton community (Keva et al., 2021).

Environmental changes and especially eutrophication have been known to change the structure of fish communities (Keva et al., 2021). It is well documented that cyprinid fish, e.g., roach (*Rutilus rutilus*) and bream (*Abramis brama*), are ultimate winners in the eutrophication in boreal lakes, whereas vendace (*Coregonus albula*) and burbot (*Lota lota*) are known to be losers (Tammi et al., 1999). However, it is not well known how dependent different fish species are on the EPA and DHA content of their prey. The ability of freshwater fish to biosynthesize longer‐chain PUFA from their precursors is reportedly better than with marine fish (Sargent et al., 1999). Nevertheless, there is a paucity of studies with different freshwater fish species are lacking. Eutrophication and browning impact on the EPA and DHA content of fish muscle are contradicting (Ahlgren et al., 1996; Keva et al., 2019; Strandberg et al., 2016; Taipale, Vuorio, et al., 2016). However, some fish species could seemingly mitigate the low nutritional quality of their prey. Ahlgren et al. (1996) found that EPA and DHA content of roach is higher in oligotrophic lakes than in eutrophic lakes, whereas they did not find a similar difference in the perch, which is in contrast to our previous finding with piscivorous perch (Taipale, Vuorio, et al., 2016). Chaguaceda et al. (2020) recently reported that the content of ARA, EPA, and DHA are strongly regulated over ontogeny in perch muscles based on their FA profiles and compound‐specific stable isotopes (Scharnweber et al., 2021). However, it is not clear how the low availability of DHA, caused by cyanobacteria blooming driven by eutrophication or climate change, impact EPA and DHA content of fish at different trophic levels.

Since European perch (*Perca fluviatilis*) have three ontogenetic dietary stages, it is an ideal fish species to evaluate eutrophication's impact on the nutritional value of the same species at different trophic levels. Perch fry eats zooplankton, from which it gradually moves to the benthos and on to fish food (Estlander et al., 2010, 2012; Haakana et al., 2007; Rask, 1986). Previously, it was found that the piscivorous (length > 20 cm) perch of oligo‐ and mesotrophic lakes contain more EPA and DHA than perch in eutrophic lakes (Gladyshev et al., 2018; Taipale, Vuorio, et al., 2016). Chaguaceda et al. (2020) suggested strong regulation of EPA and DHA in perch muscle.

Here, (H1), we hypothesized that long‐term cyanobacteria blooms by agricultural eutrophication increases the biomasses of phytoplankton, zooplankton, and fish communities, but also changes the structure of plankton and fish communities. We assumed that long‐term cyanobacterial bloom decreases the biomass of EPA‐ and DHA‐synthesizing phytoplankton taxa, favors small cladoceran over copepods, and increases the number of cyprinids over percids fish. Secondly, we hypothesized (H2) that the nutritional quality of seston is decreased by lake trophic status (Keva et al., 2021; Lau et al., 2021; Müller‐Navarra et al., 2004; Taipale, Vuorio, et al., 2016). We also assumed that this decrease in the nutritional quality of primary producers is reflected at different trophic levels via changes in the nutritional quality of their prey. Finally, we assumed (H3) that consumers try to compensate for their lower nutritional quality of prey by more efficient trophic retention and biosynthesis of physiological essential PUFA.

## 2 MATERIALS AND METHODS

### 2.1 Study area

The research material was collected during the summer of 2017 from mesotrophic Lake Pyhäjärvi and eutrophic Lake Köyliönjärvi, which are both located in southwest Finland, as shown in Table 1. Weather conditions are similar for these two lakes, which can be seen in equal surface temperature during the 2000s (PERMANOVA: Pseudo‐F_1,157_ = 0.33, *p* = .578). However, these two lakes differ in their productivity (PERMANOVA: Pseudo‐F_1,129_ = 190.4, *p* = .001) and nutrients (PERMANOVA for TP and TN: Pseudo‐F_1,181/185_ = 475/622, *p* = .001) based on measurements between 2000 and 2017 (Hertta database, Finnish Environmental Centre). Based on total phosphorus and chlorophyll concentration, Lake Köyliönjärvi can be considered a eutrophic or hyper‐eutrophic lake, whereas Lake Pyhäjärvi can be considered to be a mesotrophic lake (Bengtsson et al., 2012). Moreover, Lake Köyliönjärvi is a shallow lake (mean depth 3 m) with the deepest point of 13 m, whereas the mean depth of Lake Pyhäjärvi is 5 m, with the deepest point being 26 m. Both lakes suffer from an overly high nutrient load from their catchments. Lake Köyliönjärvi usually experiences large cyanobacterial blooms in summer, which temporarily declined in the 1990s due to fish removal (Sarvala et al., 2000). Lake Pyhäjärvi has been subjected to a variety of water protection measures since the 1980s, thereby decelerating the lake's eutrophication development (Ventelä et al., 2007, 2016). In the 2000s, climate change affected the phytoplankton community, and cyanobacteria blooms have become more frequent in Lake Pyhäjärvi (Deng et al., 2016). This development will be further accelerated in future based on the modeling (Pätynen et al., 2014).

**TABLE 1:** Total phosphorus, nitrogen, chlorophyll, turbidity, Secchi depth. and temperature for mesotrophic Lake Pyhäjärvi and eutrophic Lake Köyliönjärvi

Parameter	Unit	Mesotrophic Lake Pyhäjärvi		Eutrophic Lake Köyliönjärvi	
		2000–2017	2017	2000–2017	2017
Total phosphorus	µg P/L	19 ± 5.2	22 ± 7.0	116 ± 36.1	77 ± 42.3
Total nitrogen	µg N/L	422 ± 50	422 ± 76	1190 ± 324	992 ± 347
Chlorophyll	µg/L	7.5 ± 3.6	8.0 ± 3.1	65.1 ± 33.7	61 ± 28.7
Turbidity	FNU	2.4 ± 1.1	2.4 ± 0.7	25.5 ± 13.9	23.0 ± 6.7
Secchi Depth	m	2.5 ± 0.6	2.3 ± 0.2	0.6 ± 0.2	0.5 ± 0.1
Temperature	°C	18.4 ± 2.6	17.0 ± 1.9	18.7 ± 2.3	17.0 ± 2.2

### 2.2 Phytoplankton and zooplankton community and fatty acid sampling

Throughout the summer months (June–August) of 2017, the water quality (Secchi‐depth, water temperature, turbidity, chlorophyll‐a, total phosphorus, phosphate phosphorus, and total nitrogen), and community composition of phyto‐ and zooplankton, and their fatty acid composition and content, were monitored. A sample of 0–5 m water column was taken with a tube sampler (model: Sormunen, volume 6.3 L) to analyze quantitatively the community composition of the phyto‐ and zooplankton. Plankton community samples were analyzed by the commercial laboratory Lounais‐Suomen vesi‐ ja ympäristötutkimus Oy, where certified persons counted phytoplankton and zooplankton samples. Physico‐chemical water samples were taken with a Limnos tube sampler (volume 2.6 L) and analyzed by the Lounais‐Suomen vesi‐ ja ympäristötutkimus Oy lab. The sample points in the lakes were selected to be in line with the environmental monitoring program of the Finnish Environmental Institute, in order to utilize the water quality material found in the Hertta database (www.syke.fi/avointieto). In total, the summer sampling campaign included six samples for Lake Pyhäjärvi and five samples for Lake Köyliönjärvi.

Polyunsaturated fatty acids (PUFA) of seston (phytoplankton) available for herbivorous zooplankton were studied by pre‐filtering seston with a 50 µm sieve and then filtering a specific amount of water through GF/F filter paper (Whatman). Sampled herbivorous cladoceran was majorly (>95%) *Daphnia* and *Bosmina* and contained random (<5%) *Chydorus*, *Ceriodaphnia*, or *Diaphanosoma*. It was used to estimate the nutritional quality of diet for planktivorous perch since herbivorous cladoceran (especially *Daphnia* together with *Bosmina*) is the major prey for planktivorous perch (Estlander et al., 2010; Ruohonen, 2006). The zooplankton sample was collected horizontally with a 50 µm plankton net and main genera were picked up with microscope glass. Surface water (0–2 m water column) was sampled with a tube sampler (model: Sormunen, volume 6.3 L) for the fatty acid composition and content analysis of seston.

### 2.3 Zoobenthos community and fatty acid sampling

In addition to seasonal phyto‐ and zooplankton sampling, zoobenthos was sampled once in the littoral zone depth of 2–3 m in late summer 2017. In both study lakes, a similar sampling procedure for one sample point was carried out with an Ekman grab. The samples were filtered by a 500 µm screen to remove the fine material and then all macroscopic zoobenthos were picked up in the laboratory. Chironomidae larvae were the only abundant group in both lake samples. According to earlier studies (not published), in Lake Pyhäjärvi at least, Chironomidae larvae form a significant part of the diet for benthivorous perch.

### 2.4 Fish community and fatty acid sampling

Fish community structure and biomasses were obtained from the national fish monitoring database (Hertta/Koekalastusrekisteri) managed by the Natural Resources Institute Finland. This study covered the years 2012, 2015, 2017, and 2020 for eutrophic Lake Köyliönjärvi. Similarly, 2009, 2012, 2015, and 2019 were covered for mesotrophic Lake Pyhäjärvi. Briefly, NORDIC multimesh survey nets (Appelberg et al., 1995) were used for gillnet sampling. Gillnet sampling followed random stratified sampling, including nets in pelagic, metalimnetic, and benthic gillnets (Olin et al., 2016), whereas gillnet sampling was done yearly during July and August. The annual number of gillnet nights were 40 for eutrophic Lake Köyliönjärvi and 56 for mesotrophic Lake Pyhäjärvi. To compare fish biomasses between lakes, we used BPUE (wet mass per unit effort = kg fish per gillnet night) (Rask et al., 2020) of individual fish species. To compare the structure of perch communities in these two lakes we used CPUE (number of fish per gillnet night) of the perch group (diet group). The perch community was divided into categories including its ontogenetic diet shift (Estlander et al., 2010; Estlander, et al., 2012), planktivorous (length: <15 cm), benthivorous (15–19 cm), and piscivorous (>19 cm). These categories relate to the main diet but planktivorous fish may also feed on benthic invertebrates, and benthivorous perch feeds on smaller fish (Amundsen et al., 2003; Estlander et al., 2010, 2012).

Perch individuals for fatty acid analysis were caught in the late summer of 2017. Perches from mesotrophic Lake Pyhäjärvi were received from professional fishers who used open‐water seine fishing and gillnets for catching fish. Perch fry were also netted from a pier. Perch in Lake Köyliönjärvi were caught using the Nordic gillnet series. Due to the rapid development of young fish, the young‐of‐the‐year perch were caught within two weeks, from both lakes, to ensure the comparison between the lakes was relevant. Fry were caught on September 12 in Lake Köyliönjärvi and September 2 and 11 in Lake Pyhäjärvi. The length, weight, and sex of each fish were determined (Table S1). Age was determined mainly by using gill‐covering bone, operculum, and, in some cases, a more precise determination was made by examining scales. Samples for fatty acid analysis were taken from the dorsal muscles and stored at −20°C until they were freeze‐dried within one month from sampling. The research material covered a total of 48 fish in Lake Pyhäjärvi and 37 fish in Lake Köyliönjärvi (Table S1). In addition to perch, five individuals of small roach (<10 cm) were obtained from both lakes to estimate if PUFA content of omnivorous fish and potential diet for piscivorous perch differ in their PUFA content.

### 2.5 Fatty acid analysis

Lipids were extracted from the freeze‐dried seston, cladocera, Chironomidae, and fish samples in Kimax borosilicate tubes with chloroform‐methanol (2:1) mixture. Fatty acids were methylated using mild sulfuric acid. Methyl esterified samples were analyzed on a Shimadzu GC‐MS‐QP2010 Ultra (Nishinokyo‐Kuwabara‐Cho, Kioto, Japan) with helium as carrier gas. Column was Zebron ZB‐FAME (35 m × 0.25 mm × 0.20 µm). The temperature of the injector was 270°C and we used a splitless injection mode (for one min). Temperatures of the interface and ion source were 250 and 220°C, respectively. Phenomenex^®^ (Torrance, California, USA) ZB‐FAME column (30 m×0.25 mm×0.20 µm) with 5 m Guardian was used with the following temperature program: 50°C was maintained for one min, then the temperature was increased at 10°C/min to 130°C, followed by 7°C/min to 180°C, and 2°C/min to 200°C. This temperature was held for three minutes, and finally, the temperature increased 10°C/min to 260°C. The total program time was 35.14 min and the solvent cut time was nine minutes. Fatty acids were identified by the retention times (RT) and using specific ions which were also used for quantification (Taipale, Hiltunen, et al., 2016). Fatty acid concentrations were calculated using calibration curves based on known standard solutions (15, 50, 100, and 250 ng) of a FAME standard mixture (GLC standard mixture 566c, Nu‐Chek Prep, Elysian, MI, USA) and using recovery percentage of internal standards. The Pearson correlation coefficient was >0.99 for each individual fatty acid calibration curve. Additionally, we used 1,2‐dinonadecanoyl‐sn‐glycero‐3‐ phosphatidylcholine (Larodan, Malmö, Sweden) and free fatty acid of C23:0 (Larodan, Malmö, Sweden) as internal standards and to calculate the recovery percentages. The fatty acid content of seston (<50 µm) was calculated based on phytoplankton carbon as described by Taipale et al. (2019). Otherwise, fatty acid content was calculated based on the dry weight of zooplankton, zoobenthos, or fish muscle.

Trophic retention of ARA, EPA, and DHA by zooplankton, Chironomidae, roach, and different ontogenetic stages of perch were calculated by the following equation (referred to as accumulation factor by Hessen & Leu, 2006):

Trophic retention = (FA_diet_/FA_consumer_) – 1, where FA_diet_ represents ARA, EPA, and DHA content (µg mg/C) of diet and FA_consumer_ cites their content in the consumers. The average diet composition for each consumer was taken from previous studies. For herbivorous zooplankton, we used seston, 0+ perch, and roach. For planktivorous perch, we used herbivorous cladoceran, whereas for benthivorous perch, we used 20% of herbivorous zooplankton and 80% of *Chironomidae*. We used 0+ perch and roach for piscivorous perch (Estlander et al., 2010; Ruohonen, 2006).

### 2.6 Bulk stable isotope analysis and trophic position

Approximately 0.6–1.2 mg of freeze‐dried seston, zooplankton, benthic invertebrates, or fish muscle sample was weighted and encapsulated to tin cups. The ^15^N/^14^N was measured using a Carlo‐Erba Flash 1112 series elemental analysis connected to a Thermo Finnigan Delta Plus Advantage isotope ratio mass spectrometer in continuous flow mode. Isotopic data are presented in standard delta notation with units per mil (‰) and relative to the Vienna Pee Dee Belemnite (VPDB) international standard. Precession and accuracy were determined through repeated measurements of an internal working standard that was found to be 0.2 and 0.3, respectively.

Trophic level (TL) of consumers (herbivorous cladocera (*Daphnia* and *Bosmina*), Chironomidae, roach, and perch) was determined by using δ^15^N values (Post et al., 2002).

$${\text{TL}}_{\text{consumer}}=\lambda +\left(\delta {}^{15}N_{\text{consumer}}‐\delta {}^{15}N_{\text{baseline}}\right)/\Delta {}^{15}N,$$

where λ refers to the trophic position of the baseline organism, δ^15^N_consumer_ nitrogen stable isotope value of a given consumer, and δ^15^N_baseline_ nitrogen stable isotope values of baseline organism (seston in our case) in study lake. Δ^15^N is a trophic fractionation factor that was set 3.4‰ per trophic level according to Post (2002). Perch were divided into planktivorous (TL < 3.6), benthivorous (TL 3.6–3.9), and piscivorous (TL > 3.9) categories based on trophic levels.

### 2.7 Estimating the herbivorous cladoceran diet

We used the measured cladoceran FA profiles to estimate relative cladoceran diet compositions (%). We used Quantitative Fatty Acid Signature Analysis in R (QFASAR) (Bromaghin, 2017; Iverson et al., 2004) with χ^2^ distance measure (Stewart et al., 2014), which is the most accurate current fatty acid‐based method for herbivorous cladoceran diet estimation (Litmanen et al., 2020). The diets were estimated with an FA profile library formed of homogeneous diet feeding experiments consisting of dinoflagellates, golden algae, cryptophytes, diatoms, green algae, euglenoids, cyanobacteria, actinobacteria, and microbes sustaining on (terrestrial) particulate organic matter/detritus (Galloway et al., 2014; Litmanen et al., 2020). The standard deviation for the diet estimates was produced with 100 sample bootstrapping in QFASAR (Table S2). The estimation was conducted with R Statistical Software v. 3.6.1 (R Core Team, 2019).

### 2.8 Statistical analysis

We used PERMANOVA (Primer 7) analysis and Bray‐Curtis similarity to compare phytoplankton, zooplankton, and fish community structure at class, genus, or species level, using lake trophic status (mesotrophic or eutrophic) and month as factors. We used the same approach to compare fatty acid composition and content of essential fatty acids in phytoplankton (seston), herbivorous cladoceran, benthic invertebrates, and perches. PERMANOVA with Euclidean distance as resemblance matrix was used for univariate analysis (Anderson et al., 2017). Non‐metric multidimensional scaling NMDS was used to separate communities’ structure, fatty acid composition, and content of essential fatty acids (Primer 7). The correlations between MDS1 and MDS2 and variables were analyzed with Spearman correlation analysis. Hierarchical Cluster analysis was used to create similarity groups in NMDS. We used bubble plots to illustrate the total biomass of phytoplankton, zooplankton, and fish communities in NMDS.

## 3 RESULTS

### 3.1 Water quality and phytoplankton community

Total phosphorus, nitrogen, chlorophyll, and turbidity were significantly higher in eutrophic Lake Köyliönjärvi than in mesotrophic Lake Pyhäjärvi and lake trophic status explained 66% of the difference (Tables 1 and 2). When using two factor analysis, trophic status explained 62% of the variance (PERMANOVA: Pseudo‐F_1,13_ = 71.3, *p* = .001) and month explained 9% of the variation of phosphorus, nitrogen, chlorophyll, and turbidity (PERMANOVA: Pseudo‐F_1,13_ (trophic status/month) = 40.6./2.9, *p* = .001/.045). The temperature of surface water was equal in both lakes and whereas Secchi depth was higher in the mesotrophic Lake Pyhäjärvi than in the eutrophic Lake Köyliönjärvi during the open water season 2017 (Table 2).

**TABLE 2:** Statistical results for PERMANOVA between mesotrophic and eutrophic lakes. % cites to the contribution, FA% to fatty acid profile, concentration to µg FA mg/L, %, QFASA to the contribution of fatty acid‐based diet estimates

Component	Df1	Df2	Pseudo‐F	P(perm)	Difference
Total nitrogen	1	13	23.8	0.003	Mesotrophic < eutrophic
Total phosphorus	1	13	15.2	0.003	Mesotrophic < eutrophic
Chlorophyll a	1	13	31.6	0.001	Mesotrophic < eutrophic
Turbidity	1	13	87.9	0.001	Mesotrophic < eutrophic
Secchi depth	1	13	179.5	0.001	Mesotrophic > eutrophic
Temperature	1	13	<0.001	1	Mesotrophic = eutrophic
Dinoflagellates (%)	1	11	3.9	0.04	Mesotrophic > eutrophic
DHA‐synth. taxa (%)	1	11	8.5	0.023	Mesotrophic > eutrophic
Total phytoplankton biomass	1	10	9.3	0.0018	Mesotrophic < eutrophic
Diatoms (biomass)	1	10	9.3	0.005	Mesotrophic > eutrophic
Green algae (biomass)	1	10	526	0.002	Eutrophic > mesotrophic
Phytoplankton biomass (class)	1	10	9.3	0.023	Mesotrophic < eutrophic
Phytoplankton biomass (genus)	1	11	6.2	0.003	Mesotrophic < eutrophic
EPA‐synth. taxa (biomass)	1	11	12.2	0.002	Mesotrophic > eutrophic
DHA‐synth. taxa (biomass)	1	11	0.002	0.95	Mesotrophic = eutrophic
Predatory cladoceran	1	11	3.5	0.042	Mesotrophic < eutrophic
BPUE	1	7	23.2	0.035	Mesotrophic < eutrophic
CPUE	1	7	28.3	0.036	Mesotrophic < eutrophic
Roach (biomass)	1	7	48.1	0.033	Mesotrophic < eutrophic
Perch (BPUE)	1	7	0.27	0.641	Mesotrophic = eutrophic
Seston (FA%)	1	12	22	0.002	Mesotrophic ≠ eutrophic
Herbivorous cladoceran (FA%)	1	12	4.4	0.001	Mesotrophic ≠ eutrophic
*Chironomidae* larvae (FA%)	1	5	20.5	0.004	Mesotrophic ≠ eutrophic
Roach (FA%)	1	8	3.2	0.059	Mesotrophic = eutrophic
Perch (FA%; all sizes together)	1	93	7.7	0.001	Mesotrophic ≠ eutrophic
Perch, young of the year (FA%)	1	22	13.4	0.001	Mesotrophic ≠ eutrophic
Perch, planktivorous (FA%)	1	22	6.5	0.003	Mesotrophic ≠ eutrophic
Perch, benthivorous (FA%)	1	26	11.2	0.001	Mesotrophic ≠ eutrophic
Perch, piscivorous (FA%)	1	13	3.1	0.024	Mesotrophic ≠ eutrophic
Sestonic EPA (concentration)	1	25	0.025	0.864	Mesotrophic = eutrophic
Sestonic DHA (concentration)	1	25	49.6	0.001	Mesotrophic > eutrophic
Golden algae (%, QFASA)	1	10	10.2	0.024	Mesotrophic > eutrophic
Crypto (%, QFASA)	1	10	13.3	0.013	Mesotrophic > eutrophic
tPOM microbes (%, QFASA)	1	10	6.2	0.034	Mesotrophic < eutrophic
Dinoflagellates (%, QFASA)	1	10	2.1	0.169	Mesotrophic > eutrophic

**FIGURE 1:**
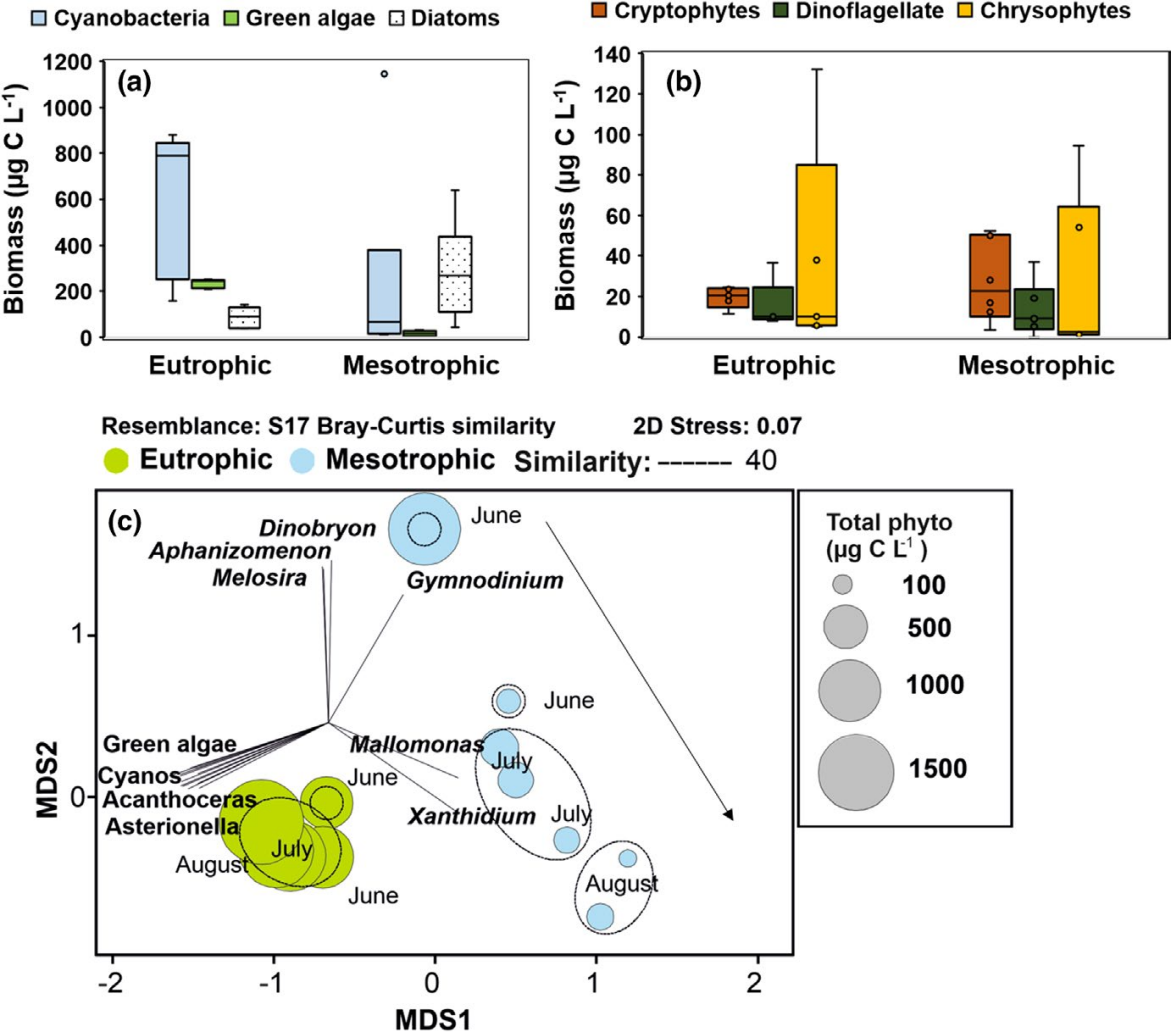
Phytoplankton biomass of the three most abundant classes (a) and three high nutritional quality classes (b) in Lake Köyliönjärvi (eutrophic) and in Lake Pyhäjärvi (mesotrophic). (c) Non‐metric multidimensional scaling output of biomasses of different phytoplankton genera. Vectors cite to the phytoplankton genus with the strong (*r* > .75, *p *< .01) Pearson correlation. Green algae cite to the genus of *Coleastrum*, *Monoraphidium*, *Oocystis*, *Pediastrum*, and *Scenedesmus*; Cyanos cite to the genus of *Chroococcus*, *Cyanodictyon*, *Microcystis*, and *Woronichia*. Bubble plots show the total phytoplankton biomass of the sample and dashed lines include samples with a 40% similarity

Cyanobacteria, diatoms, and green algae were percentually the three most abundant taxa in eutrophic Lake Köyliönjärvi, whereas cyanobacteria and diatoms were percentually the most common classes in Lake Pyhäjärvi. The contribution of dinoflagellates during summer months was significantly higher in Lake Pyhäjärvi than in Lake Köyliönjärvi, but otherwise, the lakes did not differ in their phytoplankton composition at class level (see Table 2). However, the contribution of DHA‐synthesizing taxa (cryptophytes, dinoflagellates, golden algae) was higher in the mesotrophic lake (24.3 ± 12% of all) than in the eutrophic lake (6.8 ± 7.7%). Total phytoplankton biomass was higher in eutrophic Lake Köyliönjärvi than in mesotrophic Lake Pyhäjärvi (see Figure 1). However, comparison at class level showed that only biomasses of green algae was higher in eutrophic Lake Köyliönjärvi than in mesotrophic Lake Pyhäjärvi, whereas diatom biomass was higher in the mesotrophic Lake Pyhäjärvi than in the eutrophic Lake Köyliönjärvi (refer to Figure 1, Table 2). Due to the higher biomass of diatoms in the eutrophic Lake Köyliönjärvi, the biomass of EPA‐synthesizing taxa was also higher, whereas the biomass of DHA‐synthesizing taxa did not differ between lakes (Table 2). According to the PERMANOVA, each lake explained 51% of the variation in phytoplankton biomasses at class level but explained only 36% of variation at the genus level (Table 2).

NMDS output showed that phytoplankton community structure varied greatly between the two lakes but varied more, during summer months, in mesotrophic Lake Pyhäjärvi than in eutrophic Lake Köyliönjärvi (Figure 1c). Moreover, similarity analysis (SIMPER) showed that the dissimilarity of phytoplankton communities at the genus level between the lakes was relatively high (89.4%). Cyanobacteria genus of *Dolichospermum* and *Microcystis* and diatom genera of *Aulacoseira* were more abundant taxa in eutrophic Lake Köyliönjärvi than in mesotrophic Lake Pyhäjärvi, whereas *Aphanizomenon* (Cyanobacteria) was more abundant in the mesotrophic than in the eutrophic lake. The closer comparison of EPA‐ and DHA‐synthesizing phytoplankton genus (SIMPER: Average dissimilarity = 78.7%) showed that *Aulacoseira*, *Acanthoceras*, *Uroglena*, *Rhodomonas*, *Cryptomonas*, and *Ceratium* were more abundant in eutrophic Lake Köyliönjärvi (explaining 61.1% of dissimilarity). Whereas *Dinobryon*, *Tabellaria*, *Fragilaria*, *Rhizosolenia*, and *Gymnodinium* were more abundant in mesotrophic Lake Pyhäjärvi (explaining 15.4% of dissimilarity).

### 3.2 Zooplankton community

**FIGURE 2:**
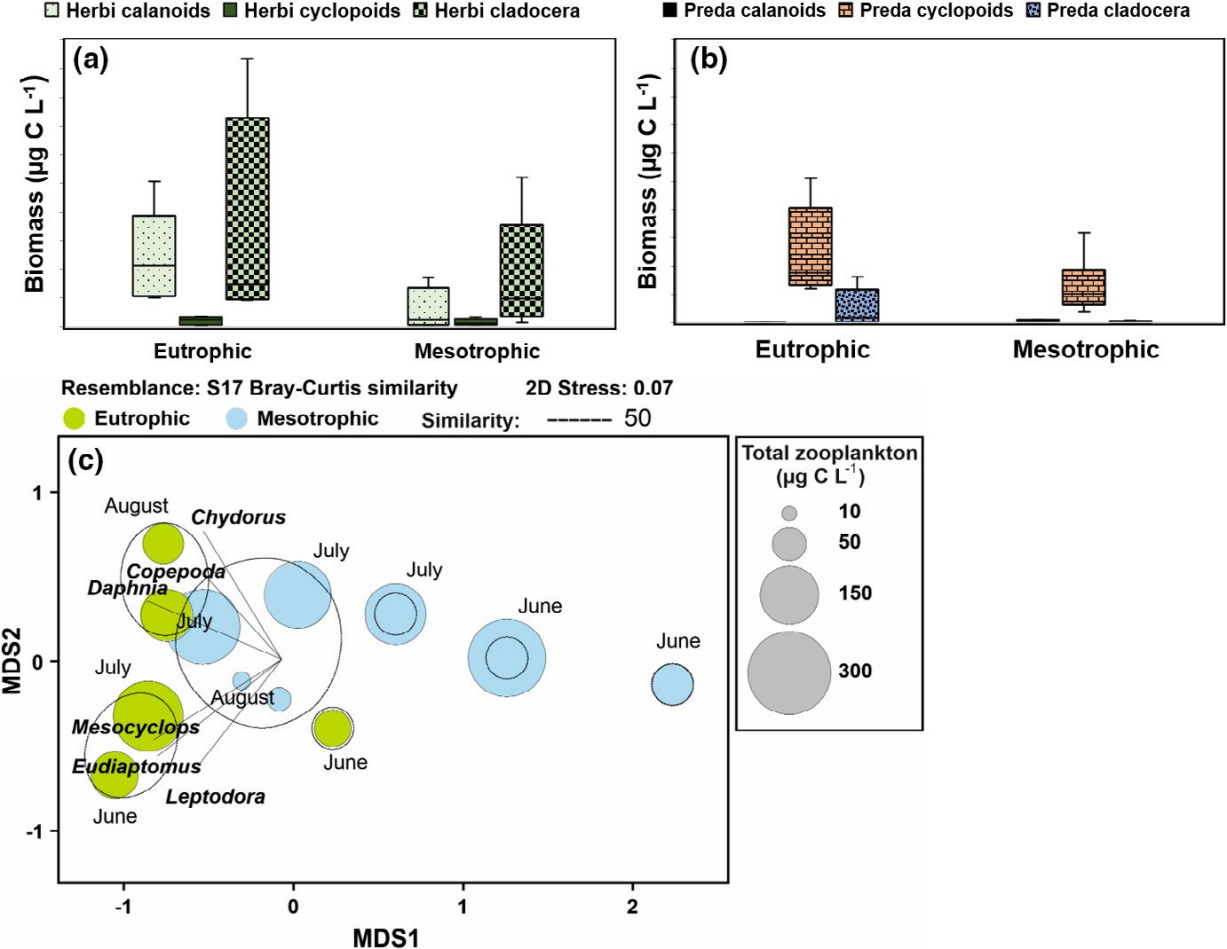
Zooplankton biomass of the (a) herbivorous calanoids (*Eudiaptomus*), cyclopoids (*Cyclopoida*) and cladocerans (*Bosmina*, *Ceriodaphnia*, *Chyrodys*, *Daphnia*, *Diaphanosoma*, *Holopedium*, *Limnosida*) and (b) predator calanoids (*Heterocope*), cyclopoids (*Eucyplops*, *Macrocyclops*, *Megacyclops*, *Mesocyclops*, *Thermocyclops*), and predatory cladocerans (*Leptodora kindtii*) in Lake Köyliönjärvi (eutrophic) and in Lake Pyhäjärvi (mesotrophic). (c) Non‐ metric multidimensional scaling output of biomasses of different zooplankton genera. Vectors cite to the zooplankton genus with the strong (*r* > .60, *p *< .01) Pearson correlation. Bubble plots show the total zooplankton biomass of the sample and dashed lines include samples with 50% similarity

NMDS output revealed changes in the zooplankton community structure between the lakes but also by the season (see Figure 2c). Two‐factor PERMANOVA of zooplankton biomasses at genus level showed the following statistical difference between the lakes (PERMANOVA (lake/month): Pseudo‐F_1,11_ = 4.3/3.3, *p* = .017/.009). Lake and month accounted for 21% and 32% of all variation, respectively. According to the similarity analysis, most of the difference between the lakes (SIMPER: average dissimilarity 67.8%) was explained by the genus of the *Chydorus*, *Eudiaptomus*, and *Mesocyclops*, which were more abundant in the eutrophic than in the mesotrophic lake. However, *Bosmina* was more abundant in the mesotrophic than in the eutrophic lake. Nevertheless, herbivorous cladoceran was the most abundant zooplankton group in both lakes (Figure 2a), of which *Chyrodus*, *Daphnia*, and *Bosmina* were the most abundant genus. *Eudiaptomus graciloides* was the only abundant herbivorous calanoid in both lakes and were the second‐most abundant zooplankton group with predator cyclopoids in both lakes. *Megacyclops*, *Mesocyclops*, and *Thermocyclops* were all abundant in mesotrophic Lake Pyhäjärvi whereas *Megacyclops* did not occur in the eutrophic Lake Köyliönjärvi. Predatory cladoceran (*Leptodoras kindtii*) was the only zooplankton group which differed statistically significantly between lakes being more abundant in eutrophic Lake Köyliönjärvi than in mesotrophic Lake Pyhäjärvi (Table 2, Figure 2b).

### 3.3 Benthic and fish communities

Our benthic invertebrate sampling was not quantitative, but our sampling in two lakes showed differences in the presence of various benthic invertebrates. Meanwhile, we found only Chironomidae in eutrophic Lake Köyliönjärvi, while our sampling of mesotrophic Lake Pyhäjärvi resulted in finding several individuals of *Asellus aquaticus*, Ephemeroptera, Oligochaeta, Megaloptera, and Plecoptera.

**FIGURE 3:**
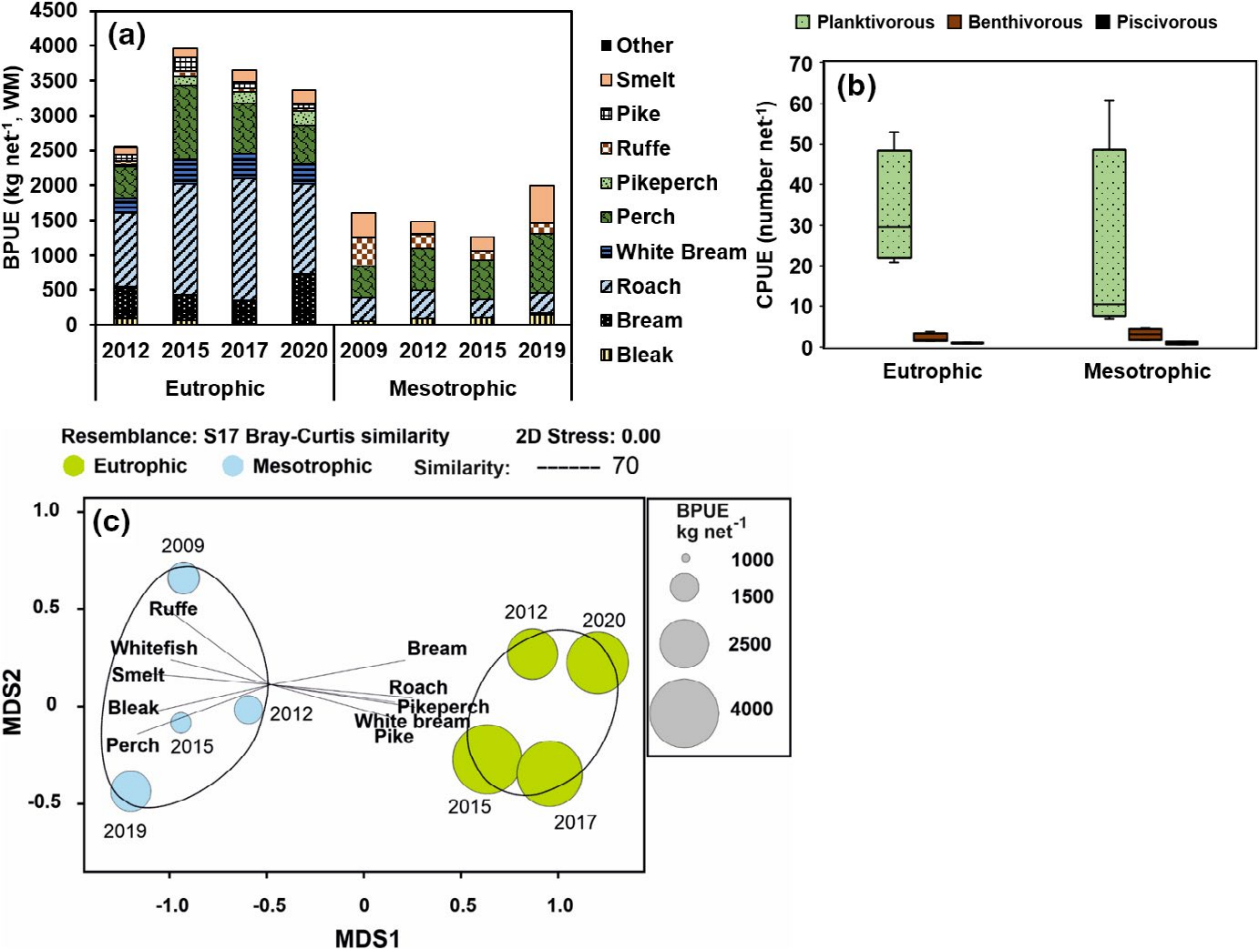
(a) Proportion of different fish species in annual total BPUE (kg/net, wet mass); (b) CPUE (number of fish net‐1) of planktivorous (0–15 cm), benthivorous (15–20 cm), and piscivorous perch in eutrophic Lake Köyliönjärvi and in mesotrophic Lake Pyhäjärvi. (c) Non‐metric multidimensional scaling output of BPUE of different fish species in Lake Köyliönjärvi and in Lake Pyhäjärvi. Vectors cite to the fish species with the strong (*r* > .60, *p *< .01) Pearson correlation. Bubble plots show the total BPUE biomass of the sample and dashed lines include samples with 70% similarity (CLUSTER)

Figure 3a showed that the Total BPUE (kg fish per gillnet night) and CPUE (number of fish per gillnet night) in 2012–2020 was higher in eutrophic Lake Köyliönjärvi than in mesotrophic Lake Pyhäjärvi (Table 2). Lake trophic status explained 83% and 79% of the variation in BPUE and CPUE, respectively. The percids were found to contribute (BPUE%) 20.6 ± 6.1% and 55.3.6 ± 5.0% of total BPUE in eutrophic Lake Köyliönjärvi and mesotrophic Lake Pyhäjärvi, respectively. Conversely, the contribution of cyprinids was higher in Lake Köyliönjärvi (66.3 ± 3.2%) than in Lake Pyhäjärvi (18.6 ± 6.5%). Roach (*Rutilus rutilus*) was the main (BPUE% = 42 ± 4%) fish species in Lake Köyliönjärvi and its biomass was statistically higher (Table 2) than in Lake Pyhäjärvi. Correspondingly, perch (*Perca fluviatilis*) was the main (BPUE% = 39 ± 7%) fish species in the Lake Pyhäjärvi; however, the BPUE of perch did not differ between lakes (Table 2). According to the SIMPER (average dissimilarity between lakes = 50.7%, Table 2) and NMDS (Figure 3c), bleak, smelt, whitefish, and ruffe were more prevalent (BPUE%) in mesotrophic Lake Pyhäjärvi, whereas pike, pikeperch, bream, and white bream were more prevalent in eutrophic Lake Köyliönjärvi.

The abundance of different ontogenetic groups of perches did not differ statistically between the lakes due to the high variation in mesotrophic Lake Pyhäjärvi (refer to Figure 3b). However, planktivorous perch contributed 91 ± 3% of all perch (CPUE%) in eutrophic Lake Köyliönjärvi, but 75 ± 17% of all perch in Lake Pyhäjärvi. Moreover, benthivorous perch contributed 20 ± 15% of all perch in Lake Pyhäjärvi, but only 6 ± 3% of all perch in Köyliönjärvi. The contribution of piscivorous perch to overall perches was similar in both lakes (~3–5%).

### 3.4 Food web structure based on fatty acids

**FIGURE 4:**
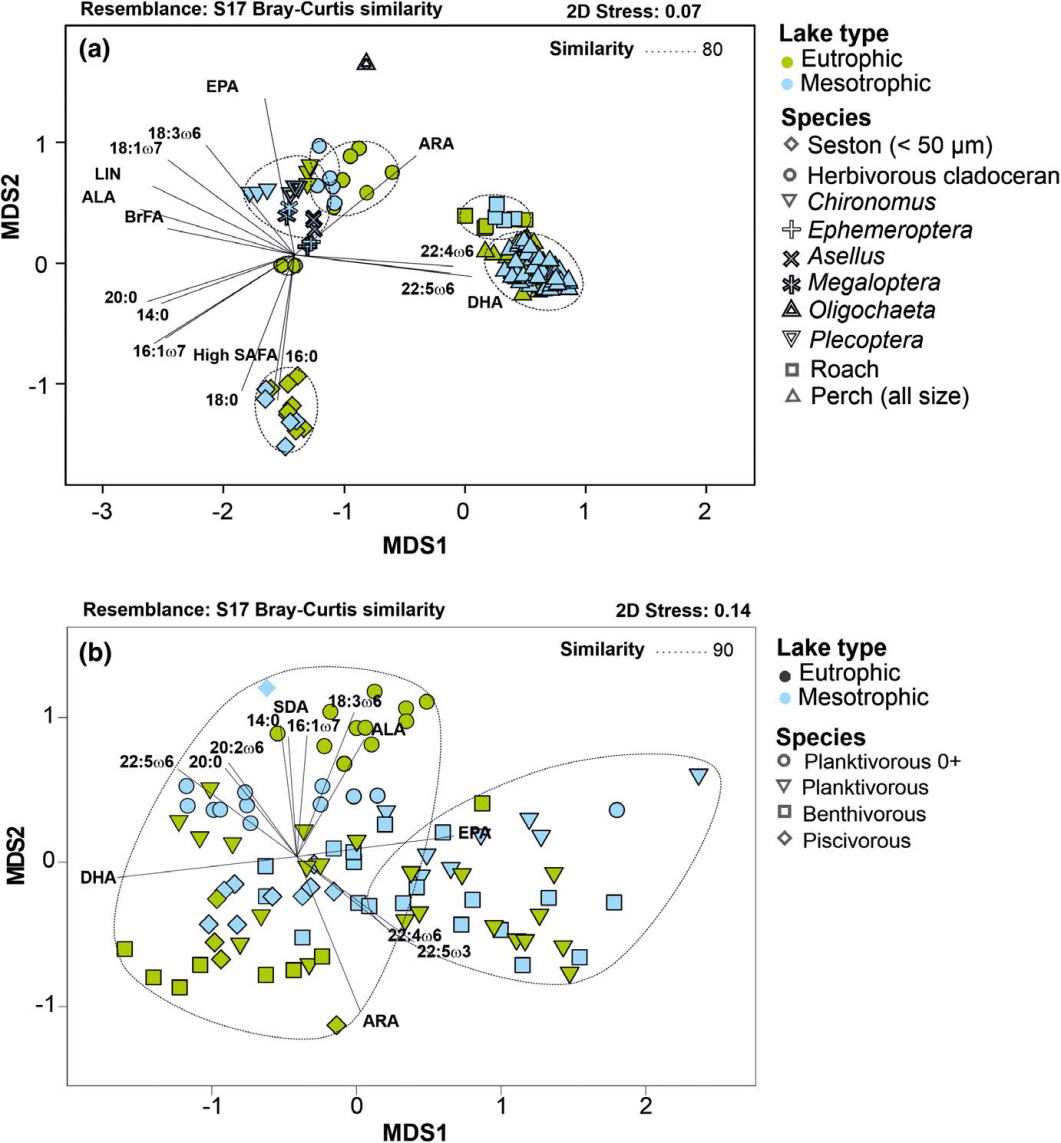
(a) Non‐metric multidimensional scaling plots of Bray Curtis similarity of fatty acid profiles (%) of seston, herbivorous cladoceran, benthic invertebrates, roach, and perch. (b) Non‐metric multidimensional scaling plots of Bray Curtis similarity of fatty acid profiles (%) of different ontogeny stages of the perch. The young‐of‐the‐year are separated from older (1+ to 2+) planktivorous perch. BrFA = branched fatty acids, High SAFA = saturated fatty acids with a carbon chain of 22–24. Dashed lines include samples with 90% similarity (CLUSTER)

According to the two‐factor PERMANOVA analysis (PERMANOVA (lake/species): Pseudo‐F_1/12145_ = 6.2/124.3, *p* = .001), lake type explained only 0.5% and species (organism) 84% of FA variation, when all fatty acid profiles of seston, zooplankton, benthic invertebrates, roach, and perch were placed in the same analysis. This same phenomenon can also be seen in NMDS output (see Figure 4). Comparison of fatty acid profiles of seston (<50 µm) between lakes showed statistical difference but lake type explained only 19% of the variation (Table 2). The dissimilarity of fatty acid profiles (SIMPER: 15.9%) was explained by the fatty acids of 18:0, 14:0, 16:1ω7c, 16:0, and ALA which together contributed 65% of the difference between lakes. Fatty acid profiles of herbivorous cladoceran (*Daphnia*, *Bosmina*) also differed between lakes and lake types and explained 29% of the variation (Table 2). According to the SIMPER, the dissimilarity between lakes was 19.2% and fatty acids of 14:0, ALA, EPA, 18:1ω7, and 16:1ω7 altogether explained 47% of the variation. Additionally, the contribution of DHA in herbivorous zooplankton was much higher in the mesotrophic than in the eutrophic lake. Fatty profiles of Chironomidae also differed significantly between lakes and the trophic status of a lake explained 85% of the variation (Table 2). The dissimilarity of fatty acid profiles of Chironomidae between lakes was 14.7% (SIMPER) and EPA, 16:1ω7, 18:1ω9, 16:0, and LIN explained most (63.9%) of the differences between lakes. The fatty acid profile of roach did not differ (P(MC) = 0.058) between lakes, whereas fatty acid profiles of perches differed between lakes (Table 2), but lake trophic status explained only 8% of the variation. Moreover, age, length, and ontogenic stage explained 34%, 86%, and 25%, respectively of variation in fatty acid profiles of all perch. A comparison of three ontogenic diet groups of perch showed a statistical difference between the two lakes in all groups. Trophic status of each lake explained 39% of the variation in fatty acid profiles of the young‐of‐the‐year and planktivorous perch and 56% in benthivorous perch, but only 7% of the variation of piscivorous perch (Table 2). Dissimilarity (SIMPER) of fatty acid profiles of different ontogenetic group perch was only 7–11%. These differences can be observed in the NMDS output as well (Figure 4b, Figure S1).

### 3.5 The content of polyunsaturated fatty acids in the food web components

**FIGURE 5:**
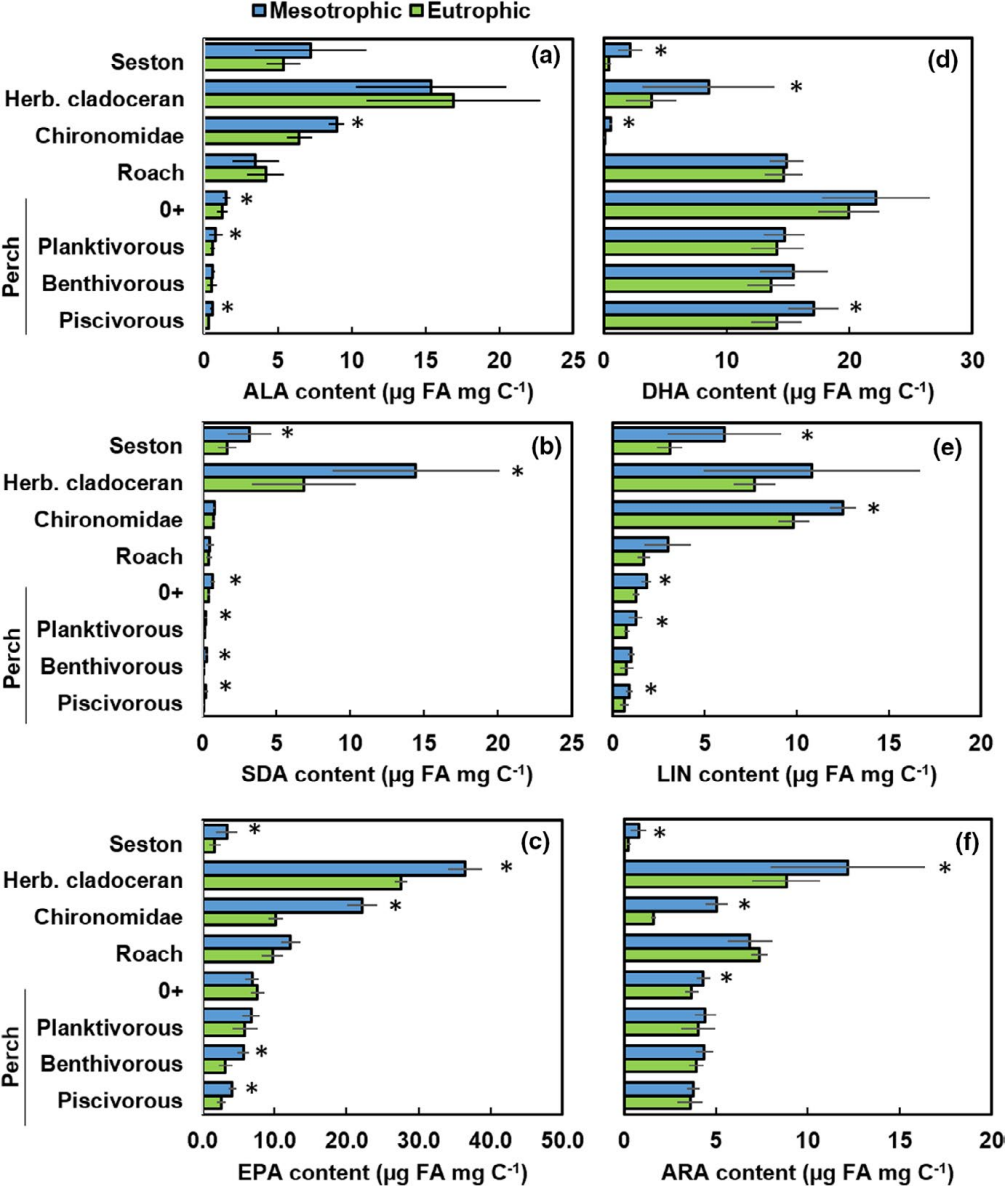
The ω‐3 (ALA, SDA, EPA, and DHA) and ω‐6 (LIN and ARA) polyunsaturated fatty acid content of food web components (seston <50 µm, herbivorous cladoceran, *Chironomidae* larvae, roach of 10–15 cm, four categories of perch) in mesotrophic Lake Pyhäjärvi and in eutrophic Lake Köyliönjärvi. Star cites to statistical difference at 0.05 level. 0+ = the young‐of‐the‐year perch

Sestonic concentration (µg FA/L) of DHA was equal in both lakes whereas the concentration of EPA was higher in the mesotrophic lake than in the eutrophic lake (Table 2). The content (µg FA/mg/C) of individual ω‐3 and ω‐6 PUFA showed different patterns across trophic levels and between lake types (Figure 5). Seasonal variation of food web components in the content of individual ω‐3 and ω‐6 PUFA was greater in the mesotrophic lake as compared to the eutrophic lake (see SD bars in Figure 5). The content of SDA (stearidonic acid), EPA, DHA, LIN, and ARA in seston (<50 µm phytoplankton) of the mesotrophic Lake Pyhäjärvi exceeded their content in the seston of eutrophic Köyliönjärvi (Pairwise PERMANOVA; Table S3, Figure 5).

**FIGURE 6:**
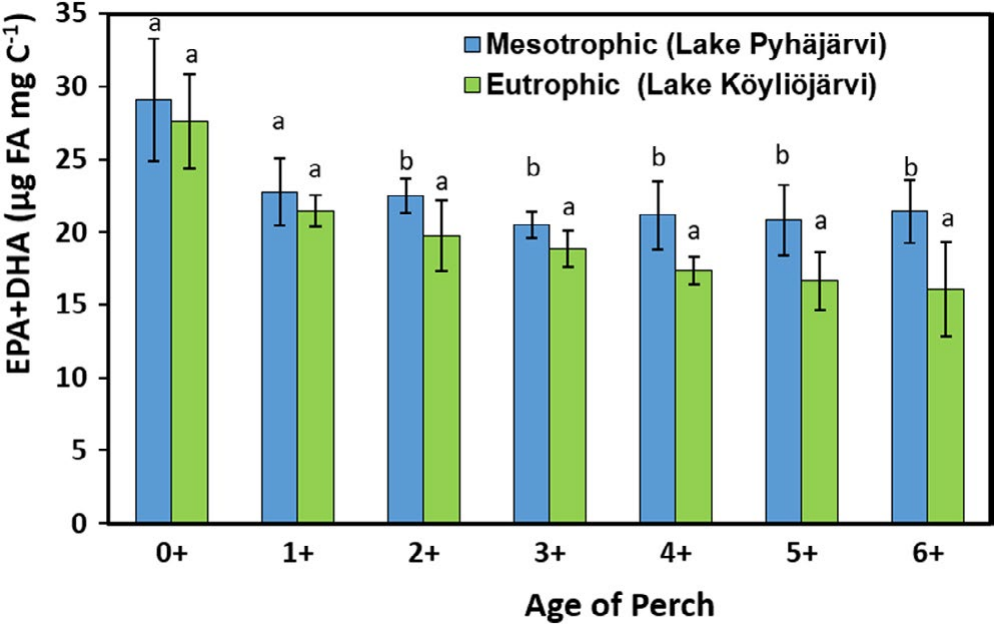
EPA + DHA content of different age group of perch in mesotrophic Lake Pyhäjärvi and eutrophic Lake Köyliönjärvi. Letter b>a cites results of pairwise PERMANOVA

Similarly, herbivorous cladoceran was found to contain more SDA, EPA, DHA, and ARA in mesotrophic Lake Pyhäjärvi than in eutrophic Lake Köyliönjärvi, whereas Chironomidae larvae contained more ALA, EPA, DHA, LIN, and ARA in the mesotrophic lake than in the eutrophic lake. Roach had equal content of all ω‐3 and ω‐6 PUFA in both lakes. The young‐of‐the‐year and planktivorous perch in the mesotrophic lake contained more ALA, SDA, and LIN than the eutrophic lake perch. Additionally, the ARA content of the‐young‐of‐the‐year was also higher in the mesotrophic lake. Both benthivorous and piscivorous perch in the mesotrophic lake contained more SDA and EPA than in the eutrophic lake. Moreover, LIN, ALA, and DHA content of piscivorous perch in the mesotrophic lake exceeded levels found in the eutrophic lake. When comparing EPA + DHA content of different age perches between the lakes, EPA + DHA content was higher in the mesotrophic than in eutrophic lake in the age groups of 2^+^ 6^+^ (PERMANOVA (lake/species): Pseudo‐F_1/12/93_ = 36.8/32.1, *p* = .001; Table S4, Figure 6).

### 3.6 Trophic retention of polyunsaturated fatty acids in mesotrophic and eutrophic lakes

**FIGURE 7:**
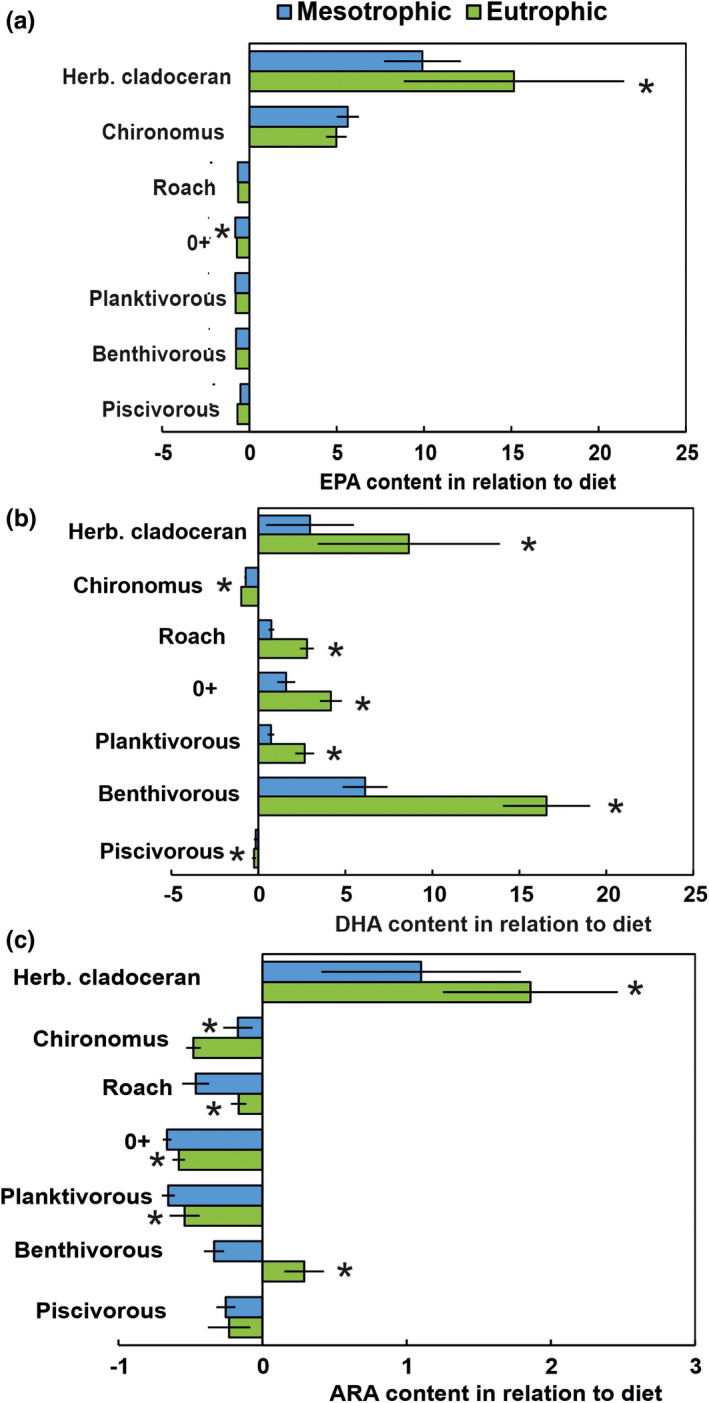
Trophic retention of EPA (a), DHA (b), and ARA (c) relating to the content of their diet. Star (*) indicates the statistical difference between mesotrophic and eutrophic lakes. See Table S3 for PERMONAVA results

Evaluation of ω‐3 and ω‐6 PUFA content of different trophic levels showed that the ALA, SDA, EPA, and ARA content was the highest in herbivorous cladocerans whereas LIN content was highest in the Chironomidae larvae, and DHA content was the highest in the young‐of‐the‐year perch. Calculations of trophic retention of ARA, EPA, and DHA between *Daphnia* and *Bosmina* and seston showed high retention of all these biomolecules in both lakes in relation to other consumers (Figure 7, Table S5). Moreover, trophic retention of these biomolecules was more efficient by *Daphnia* and *Bosmina* in the eutrophic lake than in the mesotrophic lake. Chironomidae larvae retained efficiently EPA, but not DHA or ARA from their diet and did not differ between the mesotrophic and the eutrophic lakes. Planktivorous roach and perch retained efficiently DHA, but not EPA or ARA from their diet in both lakes. Trophic retention of DHA was higher in the planktivorous roach and perch in the eutrophic in comparison to the mesotrophic lake. We found that the highest trophic retention of DHA for benthivorous perch in the eutrophic lake was 2–3 times higher than in the mesotrophic lake. Piscivorous perch have a similar amount of EPA, DHA, and ARA with their prey and thus did not retain EPA, DHA, or ARA from their diet.

### 3.7 Trophic position of consumers based δ15N value of primary producers

The δ^15^N value of primary producers was 2.7 ± 1.0‰ in mesotrophic Lake Pyhäjärvi and 4.6 ± 0.9‰ in eutrophic Lake Köyliönjärvi during sample summer months. Based on these primary producer values, the trophic position values for herbivorous cladoceran were 1.5 ± 0.1 (δ^15^N = 6.2 ± 0.5‰) and 2.2 (δ^15^N = 8.7‰) for mesotrophic and eutrophic lakes, respectively. For Chironomidae larvae, values were 2.1 ± 0.1 (δ^15^N = 8.2 ± 0.4‰) in the mesotrophic lake and 1.8 (δ^15^N = 7.3‰) in the eutrophic lake. Roach trophic position was 3.3 ± 0.1 (δ^15^N = 11.0 ± 0.3‰) in the mesotrophic lake and 3.5 ± 0.1 (δ^15^N = 10.9 ± 0.2‰) in the eutrophic lake. The trophic position of planktivorous, benthivorous, and piscivorous perch was 3.3 ± 0.1 (δ^15^N = 10.4 ± 0.5‰), 3.7 ± 0.1 (δ^15^N = 12.0 ± 0.5‰), and 4.1 ± 0.1 (δ^15^N = 13.2 ± 0.4‰) for the mesotrophic lake, and 3.5 ± 0.1 (δ^15^N = 13.1 ± 0.3‰), 3.8 ± 0.1 (δ^15^N = 14.2 ± 0.1‰), and 4.1 ± 0.1 (δ^15^N = 15.1 ± 0.3‰) for the eutrophic lake.

**FIGURE 8:**
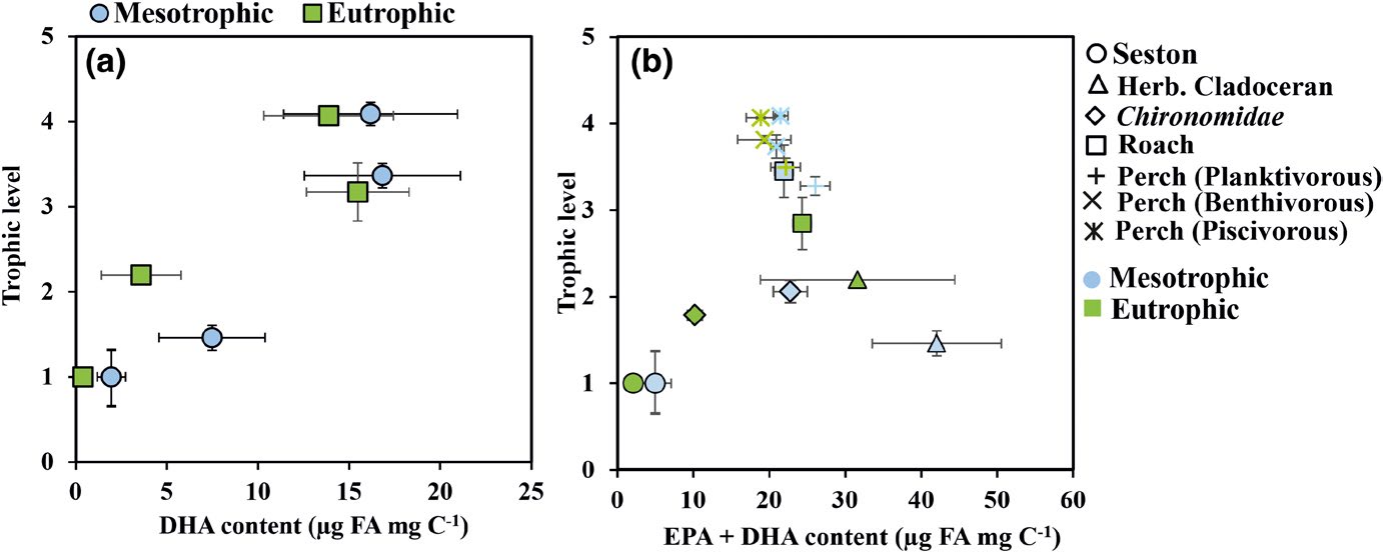
(a) The accumulation of DHA (µg FA mg/C) in the pelagic food web of the mesotrophic and the eutrophic lakes at different trophic levels (phytoplankton ‐ zooplankton ‐ planktivorous perch and roach ‐ piscivorous perch). (b) The accumulation of EPA + DHA (µg FA mg/C) at different trophic levels

The comparison of DHA accumulation at different trophic levels between mesotrophic eutrophic lakes showed great differences at first (phytoplankton) and second (herbivorous zooplankton) trophic levels (Figure 8a). On the other hand, DHA content was similar at the third trophic level (planktivorous perch and roach). Nevertheless, DHA content differed at the fourth trophic level (piscivorous perch) between mesotrophic and eutrophic lakes. The comparison of EPA + DHA content of different trophic levels showed slightly higher EPA + DHA content at first and second trophic levels of the mesotrophic lake when compared with the eutrophic lake (Figure 8b).

### 3.8 Evaluation of herbivorous cladoceran diet and transfer of EPA and DHA

The herbivorous cladoceran diets estimated with a fatty acid‐based method (QFASAR χ^2^) differed between the two lakes (Figure S2). Dinoflagellates, cryptophytes, and microbes on tPOM formed >75% of herbivorous cladoceran diet in the eutrophic lake, whereas the diets in mesotrophic Lake Pyhäjärvi also included golden algae and diatoms. Lake type accounted for 50% of the difference, and the month explained 28% of the difference between lakes (PERMANOVA (lake/month): Pseudo‐F_1/2,10_ = 14.5/4.0, P(perm) = 0.011/0.025). The contribution of golden algae in the diet of herbivorous cladoceran was statistically significantly higher in the mesotrophic lake, whereas cryptophytes and microbes on tPOM were more abundant in the diet of cladoceran in the eutrophic lake (Table 2).

Pearson regression analysis showed significant positive relation only between EPA content of herbivorous cladoceran and seston, but not with any other ω‐3 or ω‐6 PUFA (Figure S3). Moreover, the EPA content of herbivorous cladoceran was best explained by the contribution of assimilated cryptophytes, golden algae, dinoflagellates, and diatoms, whereas DHA content was best explained by the contribution of dinoflagellates and golden algae (Figure S3).

## 4 DISCUSSION

In this study, we found a clear impact of agricultural eutrophication on the structure of plankton and fish communities, but the effect on the nutritional value of food web components varied at different trophic levels. Presumably, total phytoplankton biomass was four times higher in the lake with agricultural eutrophication than in the mesotrophic lake. The increase in phytoplankton biomass caused by eutrophication is well documented in temperate and boreal lakes (Taipale et al., 2019; Taipale, Vuorio, et al., 2016; Vollenweider et al., 1974). Cyanobacteria were abundant throughout the summer months in the eutrophic lake; however, since cyanobacteria (*Aphanizomenon*) were abundant for a short period during early summer in the mesotrophic lake, the average cyanobacteria biomass for the whole summer period did not differ between lakes. However, mesotrophic and eutrophic lakes differ in their cyanobacteria species composition due to the different TP thresholds for different species (Vuorio et al., 2020). Seasonal succession of different phytoplankton species was higher in the mesotrophic lake than in the agricultural eutrophic lake. However, lakes did not differ by the contribution or the biomass of EPA‐synthesizing phytoplankton taxa, but only in the DHA‐synthesizing taxa. This was also evidenced in the higher DHA content per phytoplankton carbon in the mesotrophic lake than in the eutrophic lake, as previously found for boreal lakes (Taipale et al., 2019). Even though phytoplankton communities in mesotrophic and eutrophic lakes did not differ at class level, seston fatty acid profiles (<50 µm) were found to differ between the lakes, reflecting changes in the available phytoplankton diet for herbivorous zooplankton. Moreover, the contribution and the content of ARA, EPA, and DHA in seston was higher in the mesotrophic lake than in the eutrophic lake, as found in prior studies (Lau et al., 2021; Müller‐Navarra et al., 2004; Taipale, Vuorio, et al., 2016). Fatty acid biomarkers for golden algae and dinoflagellates in <50 µm seston suggested a higher availability of these algae for herbivorous zooplankton in the mesotrophic lake than in the eutrophic lake.

Considering our hypothesis, calanoids (*Eudiaptomus graciloides*) was equally abundant in both lakes and the biomass of herbivorous cladoceran or calanoids did not differ between lakes, suggesting that they obtained enough essential biomolecules for sustaining such a high biomass; this observation is also congruent with previous findings (Havens & Beaver, 2011). Here, we focused on herbivorous cladoceran (*Daphnia* and *Bosmina*) whose nutritional value was higher in the mesotrophic lake than in the eutrophic lake throughout the summer months. This was mainly attributed to the differences in the feeding behavior between lakes. Herbivorous cladoceran (*Daphnia* and *Bosmina*) assimilated more golden algae and dinoflagellates in the mesotrophic lake than in the eutrophic lake resulting a higher EPA and DHA content. Therefore, our study confirms that golden algae and dinoflagellates are important sources of EPA and DHA for the pelagic food web as previously suggested (Taipale, Vuorio, et al., 2016). In this current study, dinoflagellates differed in the species composition between lakes: *Ceratium* was the main species in eutrophic Lake Köyliönjärvi whereas *Gymnodinium* was the main species in mesotrophic Lake Pyhäjärvi. *Ceratium* is a large dinoflagellate, typical of eutrophic lakes, whereas *Gymnodinium* is small sized and typical of oligo‐ and mesotrophic lakes (Willén, 2003). *Ceratium* was the only abundant dinoflagellate species in Lake Köyliönjärvi, but because it is too large for herbivorous cladoceran (Santer, 1996), it is unclear what dinoflagellate species *Daphnia* and *Bosmina* were actually feeding on.

Diatoms were relatively common taxa in both lakes, but *Daphnia* and *Bosmina* did not consume them in high proportion, which may be the result of their filamentous form or low nutritional quality due to the growth stage (Diekmann et al., 2009; Jónasdóttir, 1994; Jónasdóttir & Kiørboe, 1996). In contrast, *Daphnia* and *Bosmina* fed on cryptophytes in both lakes; these are both common in eutrophic lakes and form a superior diet for herbivorous zooplankton (Lepistö & Rosenström, 1998; Peltomaa et al., 2017; Taipale, Galloway, et al., 2016). According to our fatty acid‐based diet estimates, herbivorous cladoceran (*Daphnia* and *Bosmina*) did not feed directly on cyanobacteria in either lake, thus confirming previous findings that cyanobacteria is a dead‐end in the energy flow and biomolecule transfer (Porter & McDonough, 1984). However, we did not study the diet of *Chydorus* which has been previously shown to feed on cyanobacteria (Tõnno et al., 2016). Nevertheless, microbes on tPOM/detritus were the main dietary component of herbivorous cladoceran in the eutrophic lake, which is consistent with the previous finding that a microbial food chain on decaying cyanobacteria bloom is the main link between cyanobacteria and zooplankton (Kluijver et al., 2012; Ventelä et al., 2002). Therefore, even though zooplankton do not directly feed on cyanobacteria, their decay seems to support a high biomass of edible microbes for zooplankton.

Though it seems unlikely, it is often mentioned in the extant literature that *Daphnia* and *Bosmina* are able to detect high nutritional quality patches (Schatz & McCauley, 2007) and can select high nutritional quality particles (Hartmann & Kunkel, 1991). In this study, we found that they overcame relatively poor availability of EPA‐ and DHA‐synthesizing phytoplankton taxa in the eutrophic lake, as we measured higher trophic retention of EPA and DHA by herbivorous zooplankton (*Daphnia* and *Bosmina*) in the eutrophic than in the mesotrophic lake. Unfortunately, we were unable to compare the impact of the lake's trophic status on the DHA content of herbivorous calanoids (*Eudiaptomus*). Calanoids feed selectively on high‐quality algae, preferably on dinoflagellates with high DHA content (Kleppel, 1993). Nevertheless, results in the phytoplankton and zooplankton interface were congruent with our hypotheses that lake trophic status can decrease the nutritional value of phytoplankton and herbivorous zooplankton.

Chironomidae larvae were found to contain very small amounts of DHA, but high amounts of EPA, which supports previous findings in many benthic invertebrates (Ahlgren et al., 2009; Goedkoop et al., 2000; Strandberg et al., 2020; Vesterinen, et al., 2021). Our result showed that eutrophication and long‐term cyanobacteria blooming affect the nutritional value of Chironomidae larvae whose EPA and DHA content was dramatically lowered in the eutrophic lake. This negative impact on the nutritional value was even greater with Chironomidae larvae than when found with herbivorous zooplankton. However, the EPA content of Chironomidae larvae can differ greatly among different species (Makhutova et al., 2017), and thus, the lower EPA and DHA content of Chironomidae larvae in the eutrophic lake could be attributed to different species. This is supported by the fact that *Chironomidae* larvae differ in their trophic position between lakes. Altogether, our results indicate that Chironomidae larvae were unable to biosynthesize EPA from ALA, which is opposite to Strandberg et al. (2020) laboratory experiment with *Chironomus* larvae.

Our data covered four different years of fish community data in the 2000s; cyprinid fish species were found to be more abundant in eutrophic Köyliönjärvi than in mesotrophic Lake Pyhäjärvi. Correspondingly, whitefish, vendace, and smelt were more abundant in mesotrophic Lake Pyhäjärvi than in eutrophic Lake Köyliönjärvi. However, in contrast to our hypothesis, the abundance of perch did not differ between lakes and perch can seemingly achieve higher biomasses in the eutrophic than in the oligo‐ or mesotrophic lakes (Keva et al., 2021).

Long‐term cyanobacteria blooming by agricultural eutrophication has a different impact on the nutritional value of fish at different trophic levels. Our results showed that lowered EPA and DHA content of herbivorous zooplankton (*Daphnia* and *Bosmina*) did not reflect in the EPA or DHA content of young‐of‐the‐year or planktivorous perch and roach. However, it is possible that planktivorous fish fed on DHA‐rich *Eudiaptomus*, thus obtaining DHA (Sarvala et al., 1998; Vesterinen, et al., 2021). Therefore, we were unfortunately unable to analyze *Eudiaptomus* from both lakes. Nevertheless, we found lower ARA content of the young‐of‐the‐year perch in the eutrophic lake than in the mesotrophic lake. Altogether, it seems that juvenile perch and roach successfully biosynthesized DHA from short‐chain ω‐3 PUFA, as found previously with one‐ and two‐year‐old perch in aquaculture circumstances (Henrotte et al., 2011). Moreover, the strong regulation of EPA and DHA in perch dorsal muscle was recently found in a mesotrophic lake (Chaguaceda et al., 2020). However, trout fries are not able to biosynthesize DHA from precursors (Taipale et al., 2018; Wirth et al., 1997). For this reason, even freshwater fish species differ in their ability to biosynthesize DHA from ALA (Ishikawa et al., 2019; Sargent, et al., 1999). Altogether, it is clear that DHA plays a key role in the early‐stage development of fish fry (Mourente et al., 1991). It also seems that juvenile perch preferred DHA biosynthesis over biosynthesis of ARA from LIN, since biosynthesis of ARA and DHA use the same enzymatic processes (Nielsen et al., 2019).

The high contribution of ALA and SDA in perch has been related to their planktivorous stage (Chaguaceda et al., 2020). The same fatty acids were also characteristic of the young‐of‐the‐year in both lakes and of perch relating to 1‐ to 2‐year‐old in the eutrophic lake. Most of the one‐ to two‐year‐old perch in the mesotrophic lake already cluster together with benthivorous perch, which may suggest that the same ages/size perch were in the different ontogenetic stages in the mesotrophic and eutrophic lake. The EPA + DHA content was lower in the benthivorous perch in the eutrophic than in the mesotrophic lake. Nevertheless, DHA content alone did not differ between lakes, which is why it is possible that during the low availability of DHA in their diet, they biosynthesized DHA from ALA as was recently suggested based on the compound‐specific isotope data (Scharnweber et al., 2021).

Fatty acid profiles and δ^15^N of piscivorous perch differed from two other ontogenetic stages, thus confirming their different feeding behavior and higher trophic position. Our result showed that agricultural eutrophication and long‐term cyanobacterial blooming has the greatest impact on the nutritional value of fish at upper trophic levels. The EPA and DHA content of the dorsal muscle of perch were higher in the mesotrophic lake than in the eutrophic lake, which agrees with our hypothesis. However, EPA + DHA content of piscivorous perch in the eutrophic lake was almost two times higher than the previous finding in piscivorous perches from four eutrophic boreal lakes (Taipale, Vuorio, et al., 2016). Since both eutrophication and browning (an increase of DOC) have negative relationships with EPA + DHA content of piscivorous perch, it is possible that eutrophication and browning have an additive negative impact on the perch nutritional value (Strandberg et al., 2016; Taipale, Vuorio, et al., 2016). In contrast to planktivorous and benthivorous perch, piscivorous perch has similar DHA content as their prey and trophic retention of DHA by piscivorous perch was lower in the eutrophic lake in comparison to the mesotrophic lake. However, it is also possible that large size females allocate dietary DHA to their gonad development and other crucial tasks (Keva et al., 2019).

## 5 CONCLUSIONS

In conclusion, our monitoring process throughout the summer months revealed that cyanobacteria blooming lasted much longer in the eutrophic lake suffering agricultural eutrophication when compared with the mesotrophic lake disturbed by global warming. The lakes differed in the biomass and structure of phytoplankton, zooplankton, and fish communities. Phytoplankton communities of these two lakes were found to differ at the genus level, resulting in differences in the nutritional value of seston and herbivorous zooplankton. However, the lowered nutritional value of prey did not decrease the EPA or DHA content of planktivorous perch or roach. Therefore, they seemingly compensated for the lowered nutritional value of prey by biosynthesizing EPA and DHA from their precursors. Despite compensated EPA and DHA content of planktivorous fish, piscivorous perch feeding on them were found to have lower EPA + DHA content in the eutrophic lake. Our study here emphasizes that long‐term cyanobacterial blooming by agricultural eutrophication can have varied consequences on the nutritional value of food web components at different trophic levels.

## CONFLICT OF INTEREST

None.

## AUTHOR CONTRIBUTION

**Sami Johan Taipale:** Formal analysis (supporting); Funding acquisition (supporting); Methodology (lead); Writing – original draft (lead). **Anne‐Mari Ventelä:** Supervision (equal); Writing – review & editing (supporting). **Jaakko Litmanen:** Methodology (supporting); Writing – review & editing (supporting). **Lauri Anttila:** Data curation (lead); Formal analysis (lead); Funding acquisition (lead); Investigation (lead); Methodology (equal); Writing – review & editing (supporting).

## SUBMISSION DECLARATION

This work is original, has not been previously published, and is not under consideration for publication elsewhere.

## Supporting information

Appendix A. Supplemental Table 1

## Data Availability

Phytoplankton, zooplankton, and fish data are available at Open access interfaces for environmental data at the Finnish Environmental Centre (www.syke.fi) and fatty acid data are available for download from Dryad (doi:10.5061/dryad.1c59zw3x1).
