# Supplementary material for: Poor nutritional quality of primary producers and zooplankton driven by eutrophication is mitigated at upper trophic levels
Source: Ecol Evol. 2022 Mar 8;12(3):e8687. doi: 10.1002/ece3.8687 (PMC8928886; doi:10.1002/ece3.8687)

**Appendix A. Supplemental Table 1**. Length, age, and gender of sampled perch in the mesotrophic and eutrophic lakes.

| **Lake** | **Lake type** | **Date** | **Length (mm)** | **Age** | **Gender** |
| --- | --- | --- | --- | --- | --- |
| Pyhäjärvi | Mesotrophic | 2.9.2017 | 50 | 0 |  |
| Pyhäjärvi | Mesotrophic | 30.8.2017 | 53 | 0 |  |
| Pyhäjärvi | Mesotrophic | 2.9.2017 | 55 | 0 |  |
| Pyhäjärvi | Mesotrophic | 30.8.2017 | 56 | 0 |  |
| Pyhäjärvi | Mesotrophic | 2.9.2017 | 57 | 0 |  |
| Pyhäjärvi | Mesotrophic | 2.9.2017 | 58 | 0 |  |
| Pyhäjärvi | Mesotrophic | 2.9.2017 | 59 | 0 |  |
| Pyhäjärvi | Mesotrophic | 2.9.2017 | 61 | 0 |  |
| Pyhäjärvi | Mesotrophic | 2.9.2017 | 63 | 0 |  |
| Pyhäjärvi | Mesotrophic | 30.8.2017 | 65 | 0 |  |
| Pyhäjärvi | Mesotrophic | 2.9.2017 | 66 | 0 |  |
| Pyhäjärvi | Mesotrophic | 11.9.2017 | 68 | 0 |  |
| Pyhäjärvi | Mesotrophic | 2.9.2017 | 70 | 1 |  |
| Pyhäjärvi | Mesotrophic | 2.9.2017 | 78 | 1 |  |
| Pyhäjärvi | Mesotrophic | 2.9.2017 | 88 | 1 |  |
| Pyhäjärvi | Mesotrophic | 11.9.2017 | 101 | 2 | ♀ |
| Pyhäjärvi | Mesotrophic | 11.9.2017 | 129 | 2 | ♂ |
| Pyhäjärvi | Mesotrophic | 24.9.2017 | 139 | 2 | ♂ |
| Pyhäjärvi | Mesotrophic | 11.9.2017 | 145 | 2 | ♂ |
| Pyhäjärvi | Mesotrophic | 11.9.2017 | 147 | 2 | ♀ |
| Pyhäjärvi | Mesotrophic | 24.9.2017 | 150 | 3 | ♂ |
| Pyhäjärvi | Mesotrophic | 11.9.2017 | 150 | 3 | ♂ |
| Pyhäjärvi | Mesotrophic | 24.9.2017 | 152 | 3 | ♂ |
| Pyhäjärvi | Mesotrophic | 11.9.2017 | 153 | 3 | ♀ |
| Pyhäjärvi | Mesotrophic | 11.9.2017 | 154 | 3 | ♀ |
| Pyhäjärvi | Mesotrophic | 11.9.2017 | 155 | 2 | ♀ |
| Pyhäjärvi | Mesotrophic | 27.8.2017 | 158 | 3 | ♂ |
| Pyhäjärvi | Mesotrophic | 11.9.2017 | 158 | 3 | ♀ |
| Pyhäjärvi | Mesotrophic | 24.9.2017 | 164 | 3 | ♂ |
| Pyhäjärvi | Mesotrophic | 24.9.2017 | 165 | 3 | ♂ |
| Pyhäjärvi | Mesotrophic | 11.9.2017 | 167 | 3 | ♂ |
| Pyhäjärvi | Mesotrophic | 11.9.2017 | 175 | 4 | ♀ |
| Pyhäjärvi | Mesotrophic | 11.9.2017 | 185 | 4 | ♂ |
| Pyhäjärvi | Mesotrophic | 24.9.2017 | 189 | 4 | ♂ |
| Pyhäjärvi | Mesotrophic | 13.9.2017 | 189 | 4 | ♀ |
| Pyhäjärvi | Mesotrophic | 24.9.2017 | 193 | 4 | ♀ |
| Pyhäjärvi | Mesotrophic | 27.8.2017 | 194 | 3 | ♀ |
| Pyhäjärvi | Mesotrophic | 27.8.2017 | 196 | 4 | ♀ |
| Pyhäjärvi | Mesotrophic | 24.9.2017 | 198 | 4 | ♀ |
| Pyhäjärvi | Mesotrophic | 27.8.2017 | 212 | 5 | ♂ |
| Pyhäjärvi | Mesotrophic | 11.9.2017 | 213 | 5 | ♀ |
| Pyhäjärvi | Mesotrophic | 12.9.2017 | 214 | 6 | ♀ |
| Pyhäjärvi | Mesotrophic | 27.8.2017 | 233 | 6 | ♀ |
| Pyhäjärvi | Mesotrophic | 27.8.2017 | 235 | 5 | ♂ |
| Pyhäjärvi | Mesotrophic | 27.8.2017 | 240 | 5 | ♂ |
| Pyhäjärvi | Mesotrophic | 27.8.2017 | 240 | 6 | ♀ |
| Pyhäjärvi | Mesotrophic | 27.8.2017 | 241 | 6 | ♀ |
| Pyhäjärvi | Mesotrophic | 27.8.2017 | 262 | 6 | ♀ |
| Köyliönjärvi | Eutrophic | 12.9.2017 | 56 | 0 |  |
| Köyliönjärvi | Eutrophic | 12.9.2017 | 60 | 0 |  |
| Köyliönjärvi | Eutrophic | 12.9.2017 | 61 | 0 |  |
| Köyliönjärvi | Eutrophic | 12.9.2017 | 62 | 0 |  |
| Köyliönjärvi | Eutrophic | 12.9.2017 | 63 | 0 |  |
| Köyliönjärvi | Eutrophic | 28.7.2017 | 82 | 1 |  |
| Köyliönjärvi | Eutrophic | 28.7.2017 | 84 | 1 |  |
| Köyliönjärvi | Eutrophic | 28.7.2017 | 85 | 1 |  |
| Köyliönjärvi | Eutrophic | 12.9.2017 | 91 | 1 |  |
| Köyliönjärvi | Eutrophic | 12.9.2017 | 92 | 1 |  |
| Köyliönjärvi | Eutrophic | 12.9.2017 | 95 | 1 |  |
| Köyliönjärvi | Eutrophic | 28.7.2017 | 95 | 1 |  |
| Köyliönjärvi | Eutrophic | 12.9.2017 | 100 | 2 | ♀ |
| Köyliönjärvi | Eutrophic | 12.9.2017 | 104 | 2 |  |
| Köyliönjärvi | Eutrophic | 12.9.2017 | 106 | 2 | ♂ |
| Köyliönjärvi | Eutrophic | 12.9.2017 | 109 | 2 | ♀ |
| Köyliönjärvi | Eutrophic | 12.9.2017 | 111 | 2 | ♀ |
| Köyliönjärvi | Eutrophic | 12.9.2017 | 111 | 2 | ♀ |
| Köyliönjärvi | Eutrophic | 12.9.2017 | 115 | 2 | ♂ |
| Köyliönjärvi | Eutrophic | 12.9.2017 | 120 | 3 | ♀ |
| Köyliönjärvi | Eutrophic | 12.9.2017 | 122 | 2 | ♀ |
| Köyliönjärvi | Eutrophic | 12.9.2017 | 126 | 3 | ♀ |
| Köyliönjärvi | Eutrophic | 12.9.2017 | 130 | 3 | ♂ |
| Köyliönjärvi | Eutrophic | 12.9.2017 | 130 | 3 | ♀ |
| Köyliönjärvi | Eutrophic | 28.7.2017 | 132 | 3 | ♀ |
| Köyliönjärvi | Eutrophic | 28.7.2017 | 135 | 3 | ♂ |
| Köyliönjärvi | Eutrophic | 28.7.2017 | 136 | 3 | ♀ |
| Köyliönjärvi | Eutrophic | 28.7.2017 | 157 | 5 | ♀ |
| Köyliönjärvi | Eutrophic | 12.9.2017 | 164 | 4 | ♀ |
| Köyliönjärvi | Eutrophic | 12.9.2017 | 168 | 4 | ♀ |
| Köyliönjärvi | Eutrophic | 12.9.2017 | 172 | 4 | ♀ |
| Köyliönjärvi | Eutrophic | 28.7.2017 | 175 | 4 | ♂ |
| Köyliönjärvi | Eutrophic | 28.7.2017 | 190 | 5 | ♀ |
| Köyliönjärvi | Eutrophic | 28.7.2017 | 193 | 5 | ♀ |
| Köyliönjärvi | Eutrophic | 28.7.2017 | 198 | 6 | ♂ |
| Köyliönjärvi | Eutrophic | 28.7.2017 | 200 | 5 | ♀ |
| Köyliönjärvi | Eutrophic | 28.7.2017 | 205 | 6 | ♂ |

**Appendix B.** **Supplemental Table 2.** Results of the QFASA diet estimation (mean proportion±sd) for *Daphnia* and *Bosmina* in mesotrophic Lake Köyliönjärvi and Eutrophic Lake Pyhäjärvi during summer 2017. tPOM cites to the terrestrial particulate organic matter including macrophyte particles, tPOMb cites to the microbes on the tPOM and marcophyte particles.

| **Date** | **Lake** | **Dino-flagellates** | **Golden Algae** | **Crypto-monads** | **Diatoms** | **Green Algae** | **Euglenoids** | **Cyano-bacteria** | **Actinobacteria** | **tPOM** | **tPOMb** |
| --- | --- | --- | --- | --- | --- | --- | --- | --- | --- | --- | --- |
| 8.6.2017 | Mesotrophic | 0.496±0.043 | 0.025±0.029 | 0.210±0.046 | 0±0 | 0±0 | 0±0 | 0±0.001 | 0.004±0.014 | 0±0.005 | 0.265±0.025 |
| 21.6.2017 | Mesotrophic | 0.380±0.036 | 0.012±0.017 | 0.248±0.036 | 0±0 | 0±0 | 0±0 | 0±0 | 0±0 | 0±0 | 0.360±0.017 |
| 11.7.2017 | Mesotrophic | 0.374±0.050 | 0.008±0.022 | 0.140±0.057 | 0±0 | 0.035±0.022 | 0±0.001 | 0.030±0.049 | 0±0.001 | 0±0.014 | 0.413±0.026 |
| 27.7.2017 | Mesotrophic | 0.212±0.058 | 0±0 | 0.238±0.075 | 0±0 | 0.084±0.037 | 0±0.001 | 0±0.003 | 0.005±0.018 | 0±0 | 0.461±0.032 |
| 18.8.2017 | Mesotrophic | 0.068±0.056 | 0±0 | 0.293±0.084 | 0±0 | 0.167±0.053 | 0±0.002 | 0.040±0.082 | 0.038±0.042 | 0±0 | 0.394±0.050 |
| 5.6.2017 | Eutrophic | 0.618±0.038 | 0.151±0.034 | 0±0.001 | 0±0 | 0±0 | 0±0.001 | 0±0 | 0.005±0.014 | 0±0 | 0.226±0.023 |
| 20.6.2017 | Eutrophic | 0.421±0.051 | 0.243±0.033 | 0.134±0.088 | 0±0 | 0.030±0.027 | 0±0.002 | 0±0 | 0.012±0.016 | 0±0 | 0.160±0.025 |
| 4.7.2017 | Eutrophic | 0.403±0.034 | 0±0 | 0.203±0.033 | 0±0 | 0±0 | 0±0.002 | 0±0 | 0±0 | 0±0 | 0.394±0.019 |
| 17.7.2017 | Eutrophic | 0.416±0.031 | 0.315±0.035 | 0±0 | 0±0 | 0.021±0.017 | 0±0.001 | 0±0 | 0±0 | 0±0 | 0.248±0.020 |
| 31.7.2017 | Eutrophic | 0.468±0.045 | 0.132±0.037 | 0±0.003 | 0±0 | 0.112±0.038 | 0±0.006 | 0±0 | 0±0 | 0±0.002 | 0.288±0.030 |
| 28.8.2017 | Eutrophic | 0.256±0.032 | 0.133±0.028 | 0±0.001 | 0.406±0.051 | 0.094±0.055 | 0.053±0.010 | 0±0 | 0.011±0.011 | 0±0.020 | 0.047±0.031 |

**Appendix C. Supplemental Table 3.** Results of pairwise PERMANOVA for the content of ω-3 or ω-6 PUFA in the food web components.

| **Fatty acid** | **Organism** | **t** | **P(perm)** | **perms** | **P(MC)** |
| --- | --- | --- | --- | --- | --- |
| DHA | Herb. cladoceran | 2.4515 | 0.009 | 996 | 0.03 |
|  | 0+ | 1.4951 | 0.151 | 996 | 0.142 |
|  | Planktivorous | 0.95097 | 0.336 | 996 | 0.342 |
|  | Benthivorous | 1.2671 | 0.22 | 561 | 0.253 |
|  | Piscivorous | 2.933 | 0.015 | 965 | 0.015 |
|  | *Chironomidae* larvae | 6.9259 | 0.113 | 10 | 0.002 |
|  | Seston | 5.3065 | 0.001 | 998 | 0.001 |
|  | Roach | 0.26024 | 0.773 | 126 | 0.8 |
| EPA | Herb. cladoceran | 2.3183 | 0.034 | 997 | 0.036 |
|  | 0+ | 1.9083 | 0.074 | 996 | 0.077 |
|  | Planktivorous | 1.9028 | 0.068 | 995 | 0.056 |
|  | Benthivorous | 5.0387 | 0.002 | 564 | 0.002 |
|  | Piscivorous | 5.4282 | 0.001 | 977 | 0.001 |
|  | *Chironomidae* larvae | 8.8538 | 0.097 | 10 | 0.002 |
|  | Seston | 3.0963 | 0.008 | 994 | 0.004 |
|  | Roach | 2.0225 | 0.073 | 126 | 0.086 |
| ALA | Herb. cladoceran | 0.64008 | 0.507 | 997 | 0.534 |
|  | 0+ | 2.3039 | 0.039 | 998 | 0.039 |
|  | Planktivorous | 2.0739 | 0.018 | 995 | 0.044 |
|  | Benthivorous | 0.50851 | 0.644 | 562 | 0.661 |
|  | Piscivorous | 3.7491 | 0.005 | 981 | 0.004 |
|  | *Chironomidae* larvae | 4.3976 | 0.114 | 10 | 0.012 |
|  | Seston | 1.5061 | 0.159 | 996 | 0.159 |
|  | Roach | 0.67666 | 0.541 | 126 | 0.505 |
| LIN | Herb. cladoceran | 1.5538 | 0.142 | 997 | 0.131 |
|  | 0+ | 5.7023 | 0.001 | 999 | 0.001 |
|  | Planktivorous | 5.5852 | 0.001 | 997 | 0.001 |
|  | Benthivorous | 1.6534 | 0.137 | 578 | 0.128 |
|  | Piscivorous | 2.7247 | 0.013 | 973 | 0.017 |
|  | *Chironomidae* larvae | 4.2015 | 0.087 | 10 | 0.015 |
|  | Seston | 2.9947 | 0.004 | 998 | 0.007 |
|  | Roach | 1.9625 | 0.108 | 126 | 0.099 |
| ARA | Herb. cladoceran | 2.6305 | 0.012 | 998 | 0.022 |
|  | 0+ | 3.9036 | 0.002 | 998 | 0.002 |
|  | Planktivorous | 1.5572 | 0.137 | 998 | 0.135 |
|  | Benthivorous | 1.7203 | 0.131 | 574 | 0.131 |
|  | Piscivorous | 0.73381 | 0.456 | 974 | 0.499 |
|  | *Chironomidae* larvae | 9.4016 | 0.097 | 10 | 0.004 |
|  | Seston | 4.1177 | 0.001 | 996 | 0.002 |
|  | Roach | 0.79775 | 0.46 | 126 | 0.451 |
| SDA | Herb. cladoceran | 3.5507 | 0.006 | 995 | 0.002 |
|  | 0+ | 4.3694 | 0.001 | 995 | 0.001 |
|  | Planktivorous | 3.6832 | 0.002 | 998 | 0.001 |
|  | Benthivorous | 2.8483 | 0.024 | 568 | 0.027 |
|  | Piscivorous | 3.45 | 0.005 | 983 | 0.004 |
|  | *Chironomidae* larvae | 0.94067 | 0.596 | 10 | 0.411 |
|  | Seston | 3.05 | 0.003 | 999 | 0.005 |
|  | Roach | 0.45507 | 0.645 | 126 | 0.646 |

**Appendix D.** **Supplemental Table 4.** Results of pairwise PERMANOVA for the DHA + EPA content (µg FA mg C^-1^) of different ages of perch between mesotrophic Lake Köyliönjärvi and eutrophic Lake Pyhäjärvi.

| **Fatty acid** | **Age Group** | **t** | **P(perm)** | **perms** | **P(MC)** |
| --- | --- | --- | --- | --- | --- |
| DHA + EPA | 0+ | 0.86629 | 0.366 | 996 | 0.38 |
|  | 1+ | 1.2678 | 0.225 | 318 | 0.243 |
|  | 2+ | 2.2881 | 0.044 | 713 | 0.05 |
|  | 3+ | 3.1707 | 0.005 | 981 | 0.008 |
|  | 4+ | 3.4095 | 0.009 | 319 | 0.005 |
|  | 5+ | 2.6442 | 0.052 | 35 | 0.04 |
|  | 6+ | 3.0973 | 0.011 | 205 | 0.007 |

**Appendix E.** **Supplemental Table 5.** Statistical results for PERMANOVA between mesotrophic and eutrophic lakes for the trophic retention of herbivorous cladoceran (*Daphnia* and *Bosmina*), *Chironomidae* larvae, roach, young-of-the-year perch (0+), planktivorous perch, benthivorous perch, and piscivorous perch. *cites Monte-Carlo p-value

| **Fatty acid** | **Organism** | **Df1** | **Df2** | **Pseudo-F** | **P(perm)** |
| --- | --- | --- | --- | --- | --- |
| EPA | Herb. cladoceran | 1 | 21 | 7.78 | **0.011** |
|  | *Chironomidae* larvae | 1 | 5 | 1.74 | 0.299 |
|  | Roach | 1 | 8 | 0.21 | 0.688 |
|  | 0+ | 1 | 22 | 48.96 | **0.001** |
|  | Planktivorous | 1 | 41 | 3.12 | 0.098 |
|  | Benthivorous | 1 | 11 | 0.03 | 0.862 |
|  | Piscivorous | 1 | 16 | 25.59 | **0.001** |
| DHA | Herb. cladoceran | 1 | 21 | 11.59 | **0.003** |
|  | *Chironomidae* larvae | 1 | 5 | 43.44 | **0.004*** |
|  | Roach | 1 | 8 | 114.46 | **0.006** |
|  | 0+ | 1 | 22 | 442.32 | **0.001** |
|  | Planktivorous | 1 | 41 | 227.05 | **0.001** |
|  | Benthivorous | 1 | 11 | 90.51 | **0.002** |
|  | Piscivorous | 1 | 16 | 3.06 | 0.09 |
| ARA | Herb. cladoceran | 1 | 21 | 7.03 | **0.014** |
|  | *Chironomidae* larvae | 1 | 5 | 22.42 | **0.009*** |
|  | Roach | 1 | 8 | 31.59 | **0.021** |
|  | 0+ | 1 | 22 | 113.31 | **0.001** |
|  | Planktivorous | 1 | 41 | 19.95 | **0.001** |
|  | Benthivorous | 1 | 11 | 109.03 | **0.002** |
|  | Piscivorous | 1 | 16 | 0.20 | 0.673 |

**Appendix F**. **Supplemental Figure 1.** Non-metric multidimensional scaling plots of Bray Curtis similarity of fatty acid profiles (%) of young-of-the-year perch (a), planktivorous perch (b), benthivorous perch (c), piscivorous perch (d), and seston, herbivorous cladoceran, benthic invertebrates, and roach concentration of phytoplankton (µg PUFA L ^−1^) (e) in the mesotrophic Lake Pyhäjärvi and the eutrophic Lake Köyliönjärvi. TN—Total Nitrogen, TP—Total Phosphorus, Temp—temperature in the epilimnion. S = summer, F = fall, Sp = Spring.


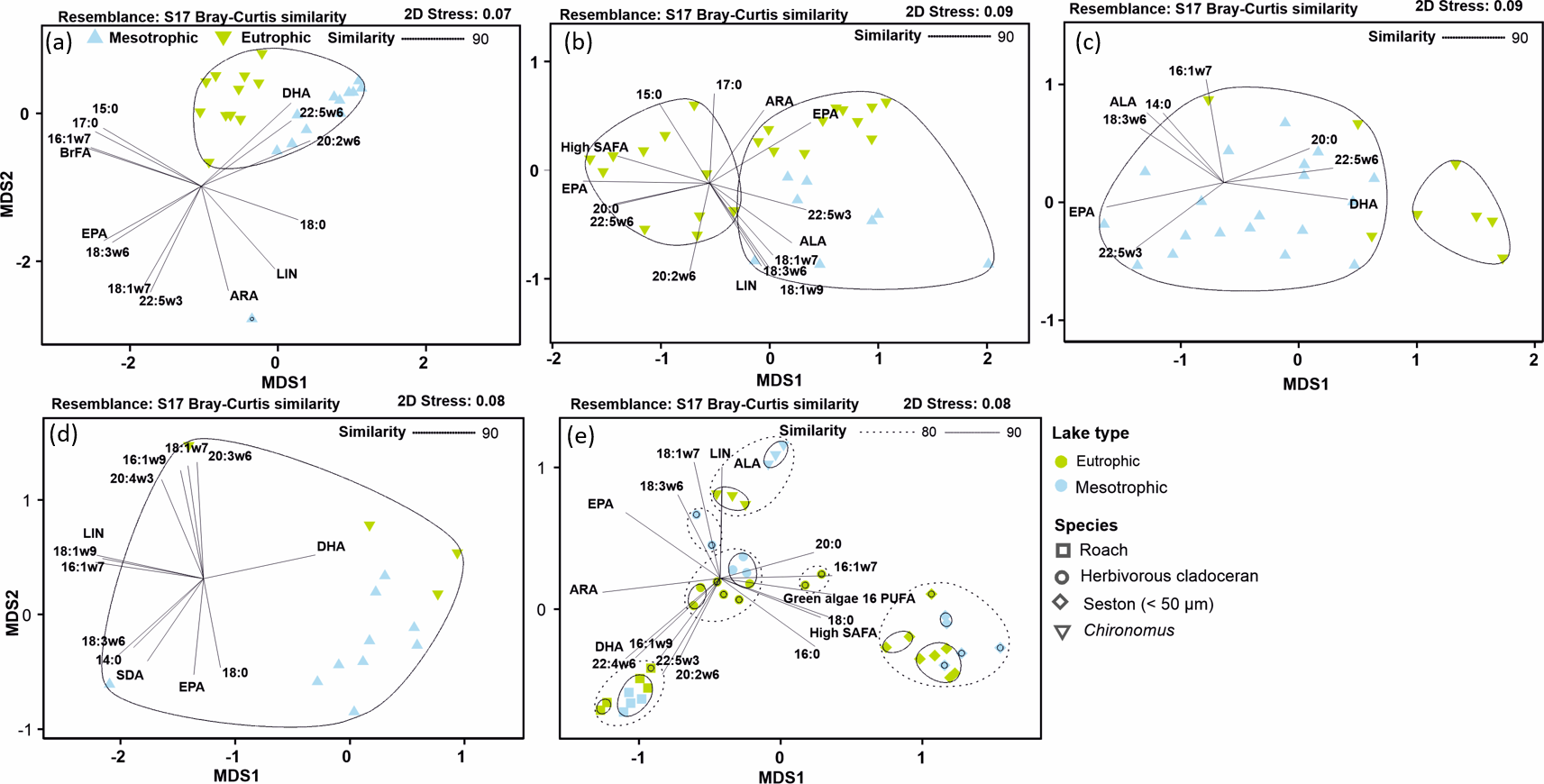


**Appendix G. Supplemental Figure 2.** Estimated diet profiles of herbivorous cladocerans in eutrophic Lake Köyliönjärvi and mesotrophic Lake Pyhäjärvi.

**

**

**Appendix H. Supplemental Figure 3.** Relationship between EPA content (µg mg^-1^ C^-1^) in herbivorous zooplankton and seston/phytoplankton. (a) The contribution of EPA-synthesized phytoplankton taxa (Cryptophytes, Diatoms, Dinoflagellates, Golden algae) in herbivorous zooplankton diet (b); and DHA content (µg mg^-1^ C^-1^) in herbivorous zooplankton; the contribution of dinoflagellates and golden algae in the herbivorous zooplankton diet.


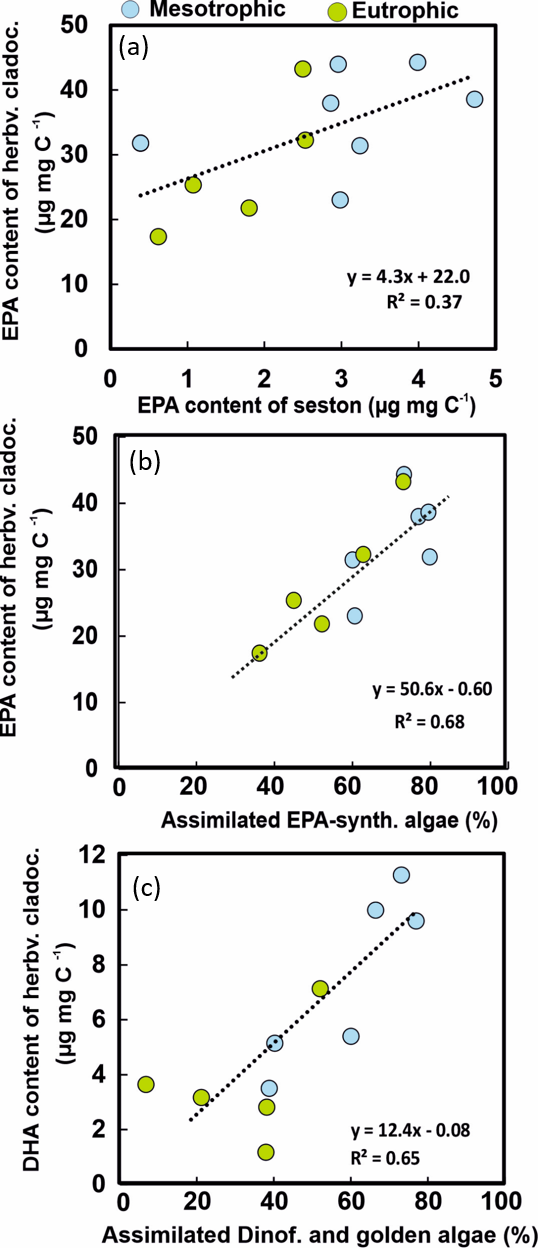

Supplement: Supplementary file 1 — Appendix A. Supplemental Table 1 [file ECE3-12-e8687-s001.docx]
